# Comorbidities, Associated Diseases, and Risk Assessment in COVID-19—A Systematic Review

**DOI:** 10.1155/2022/1571826

**Published:** 2022-10-31

**Authors:** Andreea Fitero, Simona Gabriela Bungau, Delia Mirela Tit, Laura Endres, Shamim Ahmad Khan, Alexa Florina Bungau, Ioana Romanul, Cosmin Mihai Vesa, Andrei-Flavius Radu, Alexandra Georgiana Tarce, Mihaela Alexandra Bogdan, Aurelia Cristina Nechifor, Nicoleta Negrut

**Affiliations:** ^1^Doctoral School of Biomedical Sciences, Faculty of Medicine and Pharmacy, University of Oradea, Oradea 410073, Romania; ^2^Department of Pharmacy, Faculty of Medicine and Pharmacy, University of Oradea, Oradea 410028, Romania; ^3^Department of Psycho-Neuroscience and Recovery, Faculty of Medicine and Pharmacy, University of Oradea, Oradea 410073, Romania; ^4^Faculty of Medicine and Pharmacy, University of Oradea, Oradea 410073, Romania; ^5^Department of Dental Medicine, Faculty of Medicine and Pharmacy, University of Oradea, Oradea 410073, Romania; ^6^Department of Preclinical Disciplines, Faculty of Medicine and Pharmacy, University of Oradea, Oradea 410073, Romania; ^7^Analytical Chemistry and Environmental Engineering Department, Polytechnic University of Bucharest, Bucharest 011061, Romania

## Abstract

It is considered that COVID-19's pandemic expansion is responsible for the particular increase in deaths, especially among the population with comorbidities. The health system is often overwhelmed by the large number of cases of patients addressing it, by the regional limitation of funds, and by the gravity of cases at subjects suffering from this pathology. Several associated conditions including diabetes, cardiovascular illnesses, obesity, persistent lung condition, neurodegenerative diseases, etc., increase the mortality risk and hospitalization of subjects suffering from COVID-19. The rapid identification of patients with increased risk of death from the SARS-CoV-2 virus, the stratification in accordance with the risk and the allocation of human, financial, and logistical resources in proportion must be a priority for health systems worldwide.

## 1. Introduction

It would have taken a few months to hit the shores of one continent from another only a century ago. Currently, depending on the circumstances, one may go to almost any location on the planet in a single day, with a flight time of about 5 hours between Europe and the USA. Conditions like cosmopolitan movability, levels of high infectivity, and the emergence of novel viruses have set a perfect platform for the spread of a pandemic.

Viruses, the latest being severe acute respiratory syndrome coronavirus 2 (SARS-CoV-2), are highly widespread pathogens associated with emerging infectious diseases. In Wuhan, a city in China's Hubei Province, a unique coronavirus was found to be the source of a number of pneumonia cases in December 2019 [1]. The virus dispersed exponentially, leading to the outbreak across China and, subsequently, a rising amount of occurrences in all nations worldwide. The World Health Organization named the condition “coronavirus disease 2019” (COVID-19) in February of 2020. [2]. The severe COVID-19 may occur, at otherwise healthy subjects regardless of age, , but it prevails mainly among people who have specific concomitant medical conditions or who are older.

Since SARS-CoV-2 has an impact on multiple organs by attaching to angiotensin-converting enzyme 2 (ACE_2_) receptor, patients with several comorbidities, namely, cardiovascular disease, diabetes, weight problem, asthma, chronic obstructive pulmonary disorder, immune deficiency, chronic renal impairment, neurodegenerative diseases have higher death risk. In addition to the heightened severity and mortality, these pre-existing medical conditions could be linked to COVID-19 since the metabolic system as well as the immune system are already in a compromised state because of these codiagnosed conditions [1, 3].

Previous studies related to other similar viral infections, such as Middle East respiratory syndrome (MERS) and severe acute respiratory syndrome (SARS), highlighted the increased risks related to poor prognosis in subjects presenting associated pathologies like hypertension, cardiac disorders, renal impairments, respiratory diseases, diabetes, pregnancy, and malignancy [4–7]. Wang et al. concluded in a single-center investigation carried on between January 1 and 28, 2020, that of the 138 COVID-19 subjects hospitalized in the Zhongnan Hospital of Wuhan University, China, 64 (46.4%) suffered from one or several associated pathologies. The most prevalent comorbid pathologies were malignancy (10/7.2%), diabetes (14/10.1%), hypertension (43/31.2%), and cardiovascular disease (20/14.5%) [8].

SARS-CoV-2 is still in an evolutionary phase, with numerous mutations [9] whose precise clinical course, severity, and complications are still not entirely comprehended. In order to identify individuals that are at higher risk to develop serious condition according to clinical, epidemiologic, and laboratory data, various evaluation methods were suggested; yet, the majority of studies assessing these mechanisms are constrained by the possibility of bias, and none have been prospectively evaluated or validated for therapeutic care [10].

In the present paper, we intend to offer an extensive perspective regarding the interactions of pre-existing comorbidities, COVID-19-associated diseases, and overall therapeutic results of patients suffering from COVID-19, by centralizing updated medical information offered by relevant articles and highlighting the value of a reliable risk assessment for optimal therapeutic management. Moreover, this narrative review evaluated recent clinical trials which may offer a comprehensive overview on various pathophysiological mechanisms, interactions between different diseases, and viral mutation processes within the framework of evidence-based medicine. The assessment regarding comorbidities and associated diseases correlated with COVID-19 infection may not only provide an improvement in biological approaches to personalized treatment and in the health condition of vulnerable population but also decrease the economic burden by establishing new practical considerations and recommendations on COVID-19 infection.

## 2. Methodology

**Figure 1:**
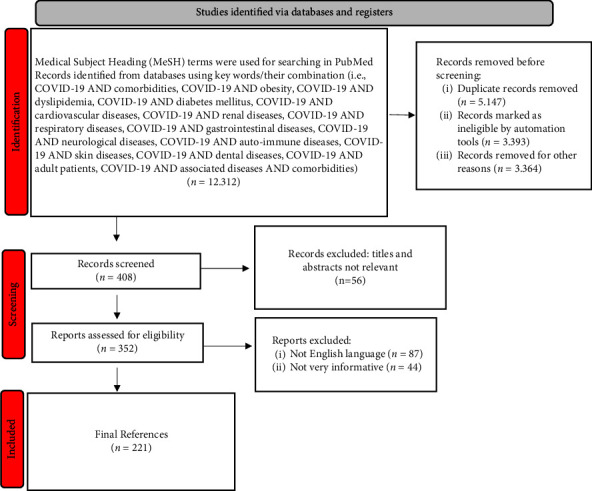
The PRISMA flowchart for the selected studies.

The present study reviews and filters articles concerning COVID-19 from 2019 to 2022, presented by a comprehensive document research related to clinical association with pre-existing comorbidities as well as associated diseases. In this regard, scientific investigation was carried out by analyzing Web of Science, National Center for Biotechnology Information, Scopus, Scientific Electronic Library Online, Centers for Disease Control and Prevention, Google Scholar databases, and Systematic Reviews from Cochrane Database. Moreover, medical subject headings (MeSH) words/phrases were used for investigating in PubMed. Overall, 220 sources were included in the bibliography to help authenticate and support the medical data in the present review (Figure 1, according to both Page et al.) [11, 12].

## 3. Comorbidities and Associated Diseases

### 3.1. Obesity

Obesity, in most of the westernized world, has become a major health concern, reaching epidemic proportions, leading to substantial morbidity [13]. It is well established that obesity affects multiple major cardiovascular disease risk factors such as HBP, diabetes mellitus type 2, and metabolic syndrome, with adverse effects on the cardiovascular system [14, 15]. Multiple studies have reported overweight as a main cause of less favorable clinical feature, prolonged hospital stays, mechanical ventilation, and a higher case fatality rate [16]. The Centers for Disease Control and Prevention (CDC) has established extreme adiposity (i.e., Body Mass Index ≥40 kg/m^2^) as a prevalent causal factor for poorer outcomes and increased mortality in patients infected with SARS-CoV-2, based on current medical data and clinical knowledge [17]. Besides, in individuals infected with SARS-CoV-2, obesity of any severity has been linked with poor prognosis [18]. Obese people are more susceptible to pneumonias with severe manifestations due to COVID-19 infection compared to individuals of average weight [19].

Elevated body mass index (BMI) is associated with decreased residual volume, functional status, and respiratory system responsiveness. Respiratory functions are imperilled in the supine position due to decreased diaphragmatic excursion in patients with increased abdominal obesity, causing ventilation to be even more problematic. Moreover, obese patients infected with SARS-CoV-2 may have a higher death rate related to elevated inflammatory cytokines linked with obesity [20]. The levels of particular proteins like chemokines or cytokines are most likely increased in the plasma of patients with obesity due to the secretion of cytokines with inflammatory activity (interleukin-10, 1, and 6 and tumor necrosis factor alpha) from adipocytes [21].

In inflamed adipose tissue, M1 macrophages, as proinflammatory cells, are over 40%, generating a variety of mediators with inflammatory effects that cause either systemic or local inflammation. Dendritic cells, neutrophils, and mast cells additionally enhance the inflammation due to the release of proinflammatory factors, resulting in chronic local and systemic inflammation [22]. Macrophages tend to move from an M2 polarized anti-inflammatory state to an M1 proinflammatory state [21].

Pluripotent stem cells from the bone marrow produce mature T lymphoid cells. Mature lymphocytes are located in several anatomical sites like the spleen, lymph nodes, and secondary lymphoid tissues, with implications for immunological processes. Lymphoid tissue function is adversely affected when the structure of lymphoid tissue is altered, leading to altered immune cell distribution, affecting T-cell activity and decreasing immunity [22–24].

Leptin, a proinflammatory adipokine secreted by adipocytes, has an effect on innate and adaptive immunity, having a role in regulating metabolic homeostasis, acting through specific receptors in the hypothalamus on the regulation of appetite [22]. Leptin resistance, found in obese patients, causes a state of hyperleptinemia, subsequently enhancing the inflammation, responsible for the unfavorable evolution of infected patients and the exacerbations of the clinical manifestations in infected overweight patients [22].

In infected individuals, the number of natural killer (NK), CD_4_^+^T, B, and CD_8_^+^T cells is reduced. T and B cells response is affected by obesity, leading to delayed immune responses [21]. Secondly, there is a rapid and sustained viral replication that is responsible for a higher viral load in obese patients [21].

Insulin resistance (IR) and excessive renin-angiotensin-aldosterone system (RAAS) activity are indicators of obesity and are associated with worse results in COVID-19 disease [25]. SARS-CoV-2 interacts with RAAS and causes infections identical to SARS-CoV-2 by utilizing the very same membrane-bound protein of the ACE_2_ receptor. In comparison to the lungs, which are organs seriously impacted by COVID-19, the adipose tissue exhibits significantly higher levels of ACE_2_ activity, suggesting that this type of tissue may be more vulnerable to the virus [26]. Furthermore, patients with obesity present an increased amount of adipose tissue, meaning higher levels of ACE_2_, which may promote viral entry and dissemination into the host cells due to the interaction of SARS-CoV-2 bound to ACE_2_.

Severe complications related to the viral infection involve widespread inflammation [22]. In a study published in 2021, Aghili et al. argued that in the case of obese patients, the elimination of the virus is longer, so these patients are more contagious because of the compromised immunological response [27].

Regarding the age implications, both young and elderly patients are vulnerable to the severe evolution of infection, but elderly patients usually present other comorbidities affecting the metabolism with a greater probability of extreme evolution.

Overweight is correlated with an enhanced death rate worldwide, while contributing to a poor quality of life. The lack of a healthy diet, with poor-quality food and a low intake of fruit and vegetables, often found in obese patients, leads to trace element and vitamin deficiencies, and secondary to severe outcomes in patients with this type of infection. Low amounts of serum vitamin D and other essential minerals like zinc, often found in obese patients, are linked in this pathology to pulmonary complications such as the acute respiratory distress syndrome [28, 29]. A sedentary lifestyle and an unhealthy diet, often encountered during the ongoing pandemic, boost the consequences of obesity, the latter posing a significant risk for serious evolution in infected patients.

### 3.2. Dyslipidemia

Until now, we knew that the presence of dyslipidemia increases the risk of cardiovascular disease, but the emerging virus SARS-CoV-2 has shown us that not only the heart and blood vessels can suffer secondary to lipid imbalance, but also respiratory diseases can have unfavorable developments in its presence.

Low-density lipoprotein (LDL) amounts are elevated in dyslipidemic patients, which, when combined with macrophages in atherosclerotic lesions, results in elevated levels of pro-inflammatory genes. Atherosclerotic plaques include a multitude of inflammatory proteins like chemokines and cytokines. Moreover, patients under statin treatment may have an increased risk of mortality and myocardial infarction. Statins could have an antiviral effect by direct antiviral action or by reducing systemic absorption of cholesterol from the target membrane.

Dyslipidemia increases the risk of severe evolution of infections with the novel coronavirus. Hariyanto and Kurniawan published in September 2020 a meta-analysis assessing 6922 infected patients in 7 experimental studies. The study claims that dyslipidemia is a contributing factor to the drastic evolution of the viral infection [30]. According to the authors, individuals suffering from dyslipidemia have elevated amounts of LDL and may interact with macrophages in atherosclerotic plaques that increase the expression of inflammatory genes. Moreover, the accumulation of LDL will form the cholesterol crystals in macrophages and that may lead to the activation of inflammation. Subsequently, inflammation generates the release of inflammatory interleukins (i.e., IL-18 and IL-1B). Cytokine storm is a serious condition of systemic inflammatory response syndrome (SIRS) that can be triggered by infection with the novel coronavirus and is caused by an abundance of inflammatory cytokines.

High-density lipoproteins (HDL) are the body's first-line defense mechanism for fighting infections, including SARS-CoV-2 infection. Small amounts of HDL will disrupt the immune response. The constant immune response triggered mostly by cytokine storm, which leads to lymphopenia and the generation of proinflammatory cytokines, is the primary cause of the severe development of SARS-CoV-2. Furthermore, the major protein of HDL is apolipoprotein I (ApoA-I). During SARS-CoV-2 infection, the composition of HDL apolipoprotein is altered by inflammation. Wang et al., in an observational study, released on 228 Chinese adults, COVID-19 patients claimed that HDL had a low level in the case of these, and a more low concentration of it was correlated with severe evolution [31].

COVID-19 is responsible for cytokine storms, and the last one, for immune‐mediated inflammatory dyslipoproteinemia. Reduced levels of LDL-C, ApoE, and HDL-C and enhanced triglyceride levels and lipoprotein oxidation, together with reduced inflammation management due to low levels of specific proresolving mediators (SPMs), are the characteristics of this occurrence. These phenomena can be partially stopped by the administration of drugs that increase ApoA‐I and HDL concentration. It is known that ApoA‐I mimetic peptides can induce inflammation reduction in human pneumocytes during influenza and control the gravity of neutrophilic airway inflammation in the case of mice affected by asthma. A high level of HDL can reinstate lipid transport function and the antioxidant function of HDL. Data available from ApoE‐deficient mice models claim that ApoA‐I and ApoE in low concentrations can induce inflammation at the lung level [32, 33].

Subsequent to the administration of fibrates, recombinant lecithin-cholesterol acyl transferase, cholesteryl ester transfer protein inhibitors, and small compounds that promote ApoA-I production, a high amount of HDL can be generated. Due to that it is recorded a decreasing platelet hyperreactivity (via cholesterol accumulation at cell level), discontinuing coagulation cascade, and downregulating of the platelet activation [34, 35].

Meta-analyses with random-effects modeling assessing the use of statins were performed using the generic inverse variance approach. A study conducted in this direction set the key outcomes as assessment of the requirement for intensive care unit (ICU) therapy and invasive mechanical ventilation (IMV) assistance but also fatality. Heterogeneous variables were used to assess each of the outcomes. Statistics from 63,537 infected patients were incorporated into a group of 28 observational investigations. The use of statins was discovered to be linked with a decreased mortality rate and the requirement for IMV, but not with the requirement for ICU management. Five distinct research subgroup analyses showed that patients who had been administered statins had even lowered mortality rates. In patients with infections by the novel coronavirus, the intake of statins was associated with a decreased need for IMV and a lower risk of mortality. Because of the risk of COVID-19 on admission, statins may not need to be stopped. To determine the clear correlation involving statin use and severe COVID-19 outcomes, more randomized controlled trials (RCTs) are needed [36, 37].

### 3.3. Diabetes Mellitus

On one hand, diabetes is the leading noninfectious and chronic disease, which over the past three decades has reached a pandemic proportion [38]. On the other hand is COVID-19, a highly infectious and acute disease. The convergence of these two pandemics has brought the medical community to its knees and forced them to inquire crucial questions about the relationship between hyperglycemia/diabetes and the magnitude of potential consequences for SARS-CoV-2. Previous research concluded that diabetes increased steadily the chance of hospitalization and substantially enhanced the risk of ICU admission and death for patients infected with the H_1_N_1_ virus [39, 40].

A fatality rate of 2.3% is revealed by statistics from the CDC, China, on more than 40,000 verified COVID-19 infections, although an abnormally high death rate of 7.3% is observed in the subset with diabetes. Furthermore, data from Italy affirmed diabetes mellitus as the second most common comorbidity linked with this viral infection, striking 33.9% of verified COVID-19 infections [41].

The majority of cardiovascular disease-related premature deaths are caused by diabetes [42]. Diabetic and obese patients are more susceptible to infections, particularly respiratory ones, since diabetes is linked to impaired immune defenses. According to Muniyappa and Gubbi, there are five main considerations for why diabetic patients are more susceptible to infection with the novel coronavirus: increased cellular binding and entrance affinities of SARS-CoV-2; decreased viral clearance; diminished T-cell activity; coexisting heart disease; and vulnerability to hyperinflammation due to the presence of cytokine storms [43].

The link between diabetes and an impaired immune system may be controlled by glycemic consequences. The discharge of interferon-gamma (IFN-*γ*) from NK cells and T-cells, as well as the expression of TNF-*α* and IL-10 from macrophages and lymphocytes seem to be suppressed by glycation [44]. The action of phagocytosis is flawed, among other processes like polymorphonuclear leukocytes mobilization and chemotaxis, due to hyperglycemia [45].

Viral replication is accentuated in patients with diabetes, either by exposing monocytes to higher glucose levels, or by activating hypoxia-inducible factor 1*α* and reactive oxygen species, secondary to glycolysis processes [46]. Viral replication causes increased IFN *γ* synthesis and activation of immune cells, causing an inflammatory process response in fat and muscle tissue, secondarily negatively influencing glucose absorption. In type 2 diabetes, the activity of NK cells was lower compared to patients with prediabetes status. In the case of diabetic patients, the value of glycated hemoglobin is a predictor of NK cell activity. Due to the reduced activity of NK cells in people with diabetes, they are more prone to acquiring severe cases of infections with the novel coronavirus, which may account for their vulnerability to the infection and bad prognosis [46].

Diabetic patients have significant concentrations of plasminogen, which decomposes the SARS-CoV-2 spike protein and boosts the virus's ability to enter cells, increasing its virulence and potential for infection [47]. Additionally, it was discovered that diabetic patients had higher amounts of inflammatory biomarkers IL-6, C-reactive protein (CRP), and D-dimer than those without the condition [48].

According to research by Jacqueline Seiglie et al. at Massachusetts General Hospital, more diabetic individuals were transferred to the ICU, required mechanical assistance, and passed away within 14 days of receiving care than patients without this specific condition [49]. In addition, COVID-19 patients with diabetes exhibit an elevated risk of thrombosis as a complication [50].

Several reports have stated that COVID-19 caused ketoacidosis leading to diabetic ketoacidosis which in turn increased the time of hospitalization for diabetic patients [51]. A specific multicenter assessment conducted in the United Kingdom described an unexpected rise in newly diagnosed type 1 diabetes in youngsters, with indicators of exposure or infection with the novel coronavirus [52]. Nonetheless, there is a dearth of data surrounding this type of disease.

COVID-19 pandemic also wreaked havoc with disruptions of care for foot ulcers in people with diabetes mellitus, leading to an increased risk of amputations [53]. Altogether, diabetic patients are indisputably at a heightened chance of serious consequences after infection with SARS-CoV-2.

A complication in both infected and diabetic patients is an imbalance in blood glucose levels, requiring adjustment of insulin doses. Inflammatory cytokine levels are related to insulin requirements [46]. Proinflammatory cytokines cause the inflammation secondary to the infection with the novel coronavirus and result in the enhancement of IR [46].

A multicenter observational study performed in 2020 on 1317 infected and diabetic French patients claimed that complications such us microvascular/macrovascular were found in 46.8%/40.8% of the cases and were responsible for the increased risk of death by 2.14/2.54 times [54]. In a study conducted in July 2020 that assessed a group of 605 infected Chinese patients requiring hospitalization but without previous diabetes, it was noted that patients with the novel coronavirus infection and fasting blood glucose values above 126 mg/dl had an increased risk of death at 28 days [55].

Treatment with oral antidiabetics such as Metformin has a protein kinase-mediated antiproliferative and immunomodulatory effect, providing protection to mice diagnosed with pneumonia [56]. It is not completely understood how antidiabetic drugs affect the probability of death from the viral infection. So far, it is known that oral administration of metformin has antiproliferative, immunomodulatory effects, associated with an anti-inflammatory, antithrombotic response, and reduction of the inflammatory cytokines (TNF and IL-6), providing protection to mice diagnosed with pneumonia, while other antidiabetic products such as pioglitazone or liraglutide increase ACE_2_ receptor expression [56–62].

Crouse et al. in a study which included 24,722 American patients infected with the novel coronavirus, claim the possibility of SARS-CoV-2 infection in a higher manner in diabetic patients, and subsequently the risk of death is much higher compared to the nondiabetic population, but the mortality was significantly lower in people who had previously been treated with metformin [62]. The patients treated with Metformin before COVID-19 had a mortality rate of 11%, slightly like the one observed in the overall population, but those without metformin administration encountered a rate of 24% [60]. Insulin administration does not appear to influence mortality in infected patients suffering from diabetes [62].

The novel coronavirus may affect insulin-producing pancreatic *β* cells (*β*-IPPs), being responsible for their apoptosis, decreased insulin levels, and increased insulin requirements in patients with COVID-19 leading to diabetic ketoacidosis and diabetes [62]. Pathogenetic processes are supported by the activity of ACE_2_ receptors along with other entry factors such as Neuropilin-1, transmembrane protein serine 2 (TMPRSS_2_), and transferrin receptor.

In male diabetic patients older than 50 years, there are more variables that raise the death rates. Additionally, diabetic men were shown to have a 2.26-fold greater mortality rate than women in the same conditions. The difference could be explained by the different constellation of gonad-corticoids, the different proportion of fatty tissue, the various inflammatory mediator levels and a plethora of innate and adaptive immune responses to diverse viral particles [62, 63].

**Figure 2:**
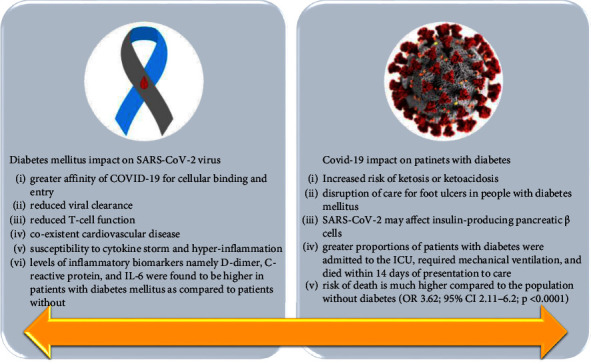
Most important characteristics/changes induced by COVID-19 in diabetes mellitus.

Figure 2 describes the most important characteristics/changes induced by the infection with the novel coronavirus in diabetes mellitus.

### 3.4. Cardiovascular Diseases

While much of the emphasis has been on respiratory complications, physicians should be aware of the cardiovascular abnormalities that can significantly affect the death rate of this viral infection. Individuals presenting established cardiovascular diseases or overburden of cardiovascular risk factors are more vulnerable to viral infections with lower survival rates [64, 65]. Li et al. pinpointed in a meta-analysis that HBP and cardio-cerebrovascular conditions are present as pre-existing in COVID-19 patients (17.1% and 16.4%), and the incidence of those conditions was twofold and threefold higher in patients admitted to ICU [66]. In a comprehensive study from China, on 44,672 confirmed COVID-19 patients, the Chinese CDC found that mortality rates were 10.5% in patients with cardiovascular disease, in contrast with just 0.9% in patients without comorbidities [67].

According to a large retrospective observational study, Gao et al. demonstrated that patients with HBP have a dramatic death risk because of the novel coronavirus infection, regardless of the administration of antihypertensive medication [68]. HBP and diabetes frequently coexist, and may synergistically facilitate adverse clinical outcomes [69]. Studies have reported the correlation between HBP and diabetes, with HBP being twice as frequent in diabetic patients as in nondiabetics [70].

The entire biomolecular process of the effect of HBP in infected patients is currently unknown, but a series of theories have been debated. The novel coronavirus penetrates by attaching to the ACE2 receptor, as was previously mentioned. The use of various antihypertensive medications, including angiotensin receptor blockers and ACE inhibitors (ACEis), may be associated with an increase in ACE2 activity at the surface of the cell, ultimately providing the virus with more “anchors” for invading cells [71, 72]. The possibility that certain patients suffering from HBP and receiving ACEis may be more vulnerable to viral infections, which might be converted into an increased risk of unfavorable evolution, cannot be exempted at this time [73]. In contrast, some researchers question if patients with HBP may experience a diminished expression of ACE2, which may generate high angiotensin II levels that increase the symptomatology of infected patients [74].

Moreover, unambiguous data supports that both systemic and pulmonary HBP are possible causes of unfavorable prognosis in pneumonia sufferers [75]. As a result, it is plausible that, in comparison to normotensive infected individuals, the coexistence of HBP and COVID-19 might interact to mutually raise the probability of a bad prognosis. The combined findings of research by Lippi et al. highlight the possibility that HBP, particularly in older people, may be associated with an increased probability of highly serious or life-threatening infection [76].

Abnormalities in echocardiography, elevated troponin levels, and symptoms of cardiac arrest were observed in a few COVID-19 patients [77]. Infection with the novel coronavirus can be the source of numerous complications, including cardiac injury. In a research study, Guo et al. proposed the mechanisms leading to such complications. Initially, a viral infection of cardiomyocytes potentially plays a pathophysiologic role in cardiovascular issues. Once the pulmonary system is affected, the virus could invade the vascular cells of the pulmonary artery and subsequently recruit immune cells which initiate an inflammatory response. The viral particles then enter the bloodstream through the pulmonary artery. The heart, which generates a large amount of ACE2, is the main place where the pulmonary circulation flows. This mechanism gives the novel coronavirus the ability to target and harm the heart [65]. Infected individuals have elevated concentrations of monocyte chemoattractant protein-1 (MCP1), IL-6, interferon gamma-induced protein 10 (IP10), IFN*γ*, and IL-1B [77]. Via direct damage to cardiomyocytes, which disrupts the cell membranes and prevents the electrical transmission of cardiac muscles, the virus can cause arrhythmia. Electrolyte abnormalities, edema, and fluid overload are further consequences of pericardial infection [78].

It was recently established that coronary artery dysfunction is linked to acute cardiovascular issues and unfavorable results in respiratory viral infections [79]. Patients infected with the novel coronavirus who have coronary artery disorders may be extremely susceptible to myocardial infarction. Because of the amplified inflammation, the built-up plaque can become unstable, leading to plaque rupture and prothrombotic milieu formation, eventually resulting in myocardial infarction [80].

Atrial fibrillation was found in 19–21% of all infected patients with the novel coronavirus, which may increase the probability of sudden death [81]. Marco et al. conducted an experimental study on 15,562 COVID-19 patients and noted that mortality in patients with pre-existing fibrillation is influenced by age, sex (higher in the case of male patients), coronary artery disease, heart failure, and diabetes mellitus. Paradoxically, young people are more exposed to mortality from atrial fibrillation, explained by the fact that the elderly usually have aggressive medication to prevent atrial fibrillation due to the high incidence of cardiac comorbidities in this age group [82]. At the base of this interaction between the viral infection and atrial fibrillation can be the disruption of the interaction between the pericyte and the endothelium, secondary synthesis of angiopoietins 1/2, basic fibroblast growth factor, and vascular endothelial growth factor, leading to inflammatory and degrading processes concerning the electrophysiology of the cell. All these are associated with hypoxemia, electrolyte disbalance, and reduction of bioavailability for ACE receptors [83].

The novel coronavirus infection can result in myocardial damage, dysrhythmias, acute coronary syndrome (ACS), venous thromboembolism, Kawasaki disease shock syndrome, heart changes, and coronary artery disease [83].

Another significant COVID-19 complication is myocarditis, which is not linked to an ischemic cause, but rather, a compounding effect of direct cell lesions, T-lymphocyte mediated cytotoxicity, and cytokine storm that comprise the pathophysiology [78]. Distinguishing myocarditis from acute coronary syndrome can be challenging. Not only are troponin values abnormal in both conditions but also electrocardiograms (ECGs) in some patients with myocarditis can mimic ACS. The anomalies in the ECG are a consequence of myocardial inflammation and consist of ST-segment deviations and abnormalities. Studies from China suggest that acute myocardial damage, marked by high cardiac markers or changes in the electrocardiogram, has led to a poor prognosis in infected patients [83]. Mortality among those suffering from the infections that present elevated troponin T was 37.5%, and it doubled among those with association between cardiovascular disease and elevated troponin T. Increased cardiac physiologic requirements, hypoxia, or direct myocardial insult have all been linked to elevated troponins in infected patients [83].

Several types of dysrhythmias have been observed in infected patients. In accordance with research by Wang et al., 44.4% of ICU patients with COVID-19 experienced dysrhythmias [5]. Hypoxia, severe inflammatory distress, and abnormal metabolism can all trigger dysrhythmias during viral infection [84].

In a report conducted in Italy on 28 patients, 24 of them had ACS with ST-segment prolongation, being the first clinical sign of infection with the novel coronavirus, before the qualitative analysis of viral ribonucleic acid (RNA), leading to the conclusion that infection may trigger ACS [83]. Atherosclerotic plaque rupture (secondary to macrophage collagenase release), coronary spasm, and the presence of small thrombi from systemic inflammation or cytokine storms may lead to this event [83]. Cardiac insufficiency was identified in 24% of the living patients and 49% of individuals who deceased, according to Chen et al. in a 2020 research of a group of 799 Asian patients infected with the novel coronavirus [85].

There is a bidirectional link between the novel coronavirus and cardiovascular disease. Firstly, the cardiac pathology influences the severity and susceptibility to the viral infection, and secondly, the inflammatory syndrome and the medication used to treat COVID-19 lead to arrhythmia, thromboembolism, acute coronary syndrome, myocarditis, or acute cardiac injury [83].

**Figure 3:**
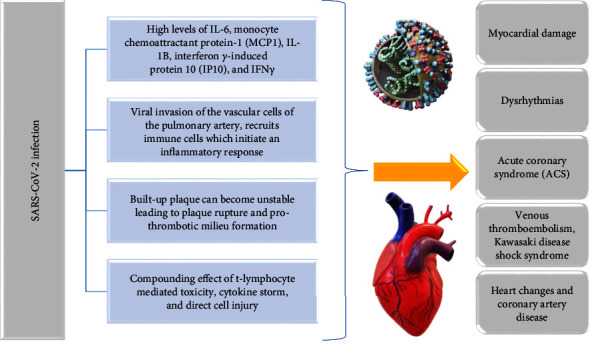
Main characteristics/changes induced by the viral infection in cardiac disorders.

Figure 3 summarizes the main characteristics/changes induced by the viral infection in heart diseases.

### 3.5. Renal Diseases

Epidemiological data indicated that although the lungs are the most affected, kidneys are also affected in severe illnesses that are associated with morbidity and mortality [86]. Lung-kidney crosstalk is established on the similarities that these two organs share and that is why a disease that affects one organ can have consequences on the other organ [87]. Respiratory failure can initiate acute kidney injury (AKI), owing to several etiologies for instance such as systemic hypoxia and acute lung injury, leading to SIRS and IMV [87]. Acute pulmonary injury caused by the infection can simultaneously harm additional organs, such as the kidneys, as a result of lung-kidney crosstalk.

The systemic hemodynamic and the neurohormonal systems are modified with the use of IMV, and its widespread use is seen in severely ill infected patients. IMV, paradoxically, can have unfavorable effects on the renal system, with this intervention being linked to a significant increase in the probability of developing AKI in ICU patients [88].

Numerous hospitalized patients die due to the infection presented by hematuria and proteinuria, which is linked to an enhanced probability of in-hospital deaths [89, 90]. A single-center, retrospective cohort study showed that AKI in hospitalized infected individuals was common and presented high mortality, particularly in patients with AKI stage 3 [91]. AKI developed in the viral infection may be caused by the synergistic interaction between the specific cytotropic action that SARS-CoV-2 induces and the systemic response that immune-mediated cytokines generate. Additionally, direct viral invasion of renal tubular cells, interstitial, or glomeruli has been postulated to cause intrinsic tissue damage. AKI, secondary to diminished renal perfusion, can be due to numerous etiologies related to this viral infection. Moreover, the novel coronavirus instigated septic shock can also provoke ischemia and hypoxia at the kidney site because of the reduction in blood perfusion.

ACE_2_ is found in high amounts in the kidneys. Based on an investigation, ACE2 expression at the renal level is similar to that in the lungs [92]. It has been reported that cytokine release syndrome induced by viral infection plays a direct role in causing acute tubular necrosis (ATN) [93] and renal tissue destruction [94]. Sepsis (found in the case of severe COVID-19 patients), renal infarction, coagulation disorders, and respiratory distress are risk factors involved in ATN. Insufficient oxygen supply and low infusion due to heart failure led to ischemic kidney damage in this viral infection [95]. Additional risk factors contributing to the development of ATN are fever and diarrhea which can lead to significant fluid loss [96].

SARS-CoV-2 also has implications for patients with renal transplants and those suffering from chronic kidney disease (CKD). A meta-analysis performed by Henry and Lippi on 1389 COVID-19 patients established a compelling independent correlation of CKD with severe forms of the viral infection [97]. Beneficiaries of kidney transplants are of indisputable concern as the use of immunosuppressive medications makes them vulnerable to COVID-19 infection.

Autopsies performed on patients with COVID-19 concluded that the damage was not only at the pulmonary, cardiac, neurological, and hepatic sites because the kidneys presented tubular necrosis, lymphocytic infiltration, and SARS-CoV-2 RNA-fragments. Regarding renal impairment, in the first phase a small number of cases with renal impairment were found (3–9%). Later an increase was observed (27–37%). The predominant stages of acute kidney damage were stage I (47%) [98].

Dialysis was used in 15% of patients with COVID-19 with renal impairment. Renal changes were present in 90% of mechanically ventilated patients and in 22% of patients who did not develop a critical form of the disease. Acute kidney damage is a risk factor for death in inpatients. The elderly, the African-American race, diabetes, HBP, cardiovascular disease, mechanically ventilated patients, and vasopressor therapy patients are all involved in acute kidney damage [98].

The biomolecular processes of renal impairment in patients infected with the novel coronavirus are apparently multifactorial and have not yet been fully elucidated. Besides direct pathophysiological mechanisms, renal impairment in connection with COVID-19 might also emerge through the systemic consequences of the viral infection. Acute prerenal lesions related to multiorgan failure, especially in patients with previous renal impairment, nephrotoxicity of the therapy used, cytokine storm and coronavirus action in the tubular cells are causes of renal impairment [98].

### 3.6. Respiratory Disease

Pulmonary failure is the main cause of mortality in infected patients because the viral infection has the strongest impact on the respiratory system [99]. The penetration of viral molecule into the ACE2-expressing pneumocytes inside the epithelial layer of the alveoli results in pulmonary stress, which manifests as diffuse alveolar destruction in the lungs [100]. A further element causing acute respiratory damage is the prolonged production of proinflammatory cytokines, resulting in a “cytokine storm” and, in turn, a strong immunological response [101]. Once innate immune cells identify viral RNA-fragments, the immune response is initiated [102]. The inflammatory mediators are released excessively and rapidly, which causes significant inflammation and lung damage that might result in pulmonary issues like acute respiratory syndrome (ARDS). Moreover, infected patients have shown postmortem clinical signs of ARDS, including pulmonary edema involving shedding of pneumocytes and the development of hyaline membranes [103].

The novel coronavirus, among other respiratory pathogens, is probable to cause asthma exacerbations since asthma is an inflammatory pulmonary condition that has been linked to the vulnerability and intensity of viral respiratory infections [104]. Moreover, multiple studies have confirmed the relatedness between viral infections and asthma exacerbations [105]. The enhanced type 2 immune mechanisms observed in asthmatics may represent the source of the patients' susceptibility to viral respiratory illnesses and exacerbations of their chronic disorder [106]. Type 2 immune responses are effectuated by T-helper cells, which produce IL4, IL-5, and IL-13. Such IL plays a part in the activation of eosinophils, production and aggregation of immunoglobulin E, and mucus production [107]. Past research has associated this immunological mechanism with diminished immune defenses, and reduced synthesis of interferons, illustrating how asthmatic individuals are susceptible to a variety of respiratory viruses [108]. On the basis of past knowledge, it was originally anticipated that asthmatics might be particularly sensitive to this viral infection. However, there have been disparities in medical information documenting the incidence of asthma in infected patients. Beurnier et al. concluded that asthma did not predominate among patients with severe symptoms in an investigation performed in France on a cohort of 768 hospitalized infected patients; only 37 of them had a history of asthma [109]. Another study conducted by Yang et al. in Korea on 7,340 infected patients states that the percentage of severe forms of the novel coronavirus infection was 6.9% in asthmatics, while for patients without asthmatic status, the rate was 4.5% [110]. Of these patients, those with non-allergic asthma had an increased risk of developing aggressive forms of infection compared to asthmatics [110].

Eosinophils are a biomarker of severity in infections with the novel coronavirus, based on the results of a study. Moreover, over 150 cells/*μ*L it is a value associated with a decreased probability of hospitalization as well as with a lower mortality due to the unfavorable evolution of COVID-19, compared to those whose eosinophils were below this value [111]. There is a variety of information that may be used to demonstrate why asthmatics do not seem to be particularly vulnerable to COVID-19. Both asthmatics and healthy volunteers were found to have similar amounts of ACE2 and TMPRSS2 gene expression [112]. This might explain why asthmatics are not more likely to get infected with the novel coronavirus.

A study by Shi T et al. on 752,867 children asserts that even though youngsters do not develop serious conditions like COVID-19, those with asthma and viral infections present a higher hospitalization rate compared to those without asthma. In addition, the hospitalization rate was considerably higher for youngsters with inadequately managed asthma than for individuals who had effectively managed asthma [113].

In addition, a current investigation found that nonallergic asthma was linked to the severity of the infection, whereas allergic asthma did not demonstrate the same results [114]. Furthermore, type 2 defense mechanisms connected to allergic asthma might aid in understanding its potential protective function in COVID-19. Blocking the release of inflammatory mediators such as IL-6 and TNF is a function of type 2 related molecules such as IL-4 and IL-13, according to experimental findings [107]. The cytokine storm condition and unfavorable prognosis in infected patients were discovered to be linked with elevated levels of these inflammatory mediators [115]. Therefore, blocking them could improve clinical outcomes for patients. The drop in eosinophil concentrations seen in infected individuals is another point for commendation. This might assist patients by compensating for dropped eosinophil concentrations during infection, considering type 2 asthma and eosinophilia are related [107].

A further chronic lung disease that may be worsened particularly by viral infections is considered the chronic obstructive pulmonary disease (COPD) [116]. Patients suffering from COPD are the most common elderly people who have other chronic diseases associated with them, and the association increases the risk of aggravation of an acute respiratory infection, against the background of an organism already weakened by existing chronic pathologies.

In a study of 10,420 Spanish patients, of which 746 had COPD, published in 2020, it was claimed that the average age of COPD and infected patients was over 70, and the mortality rate in their cases was 38.3% vs 19.2% (without COPD). Predisposing factors for a rise in SARS-CoV-2 infection-related deaths included males, HBP, heart failure, chronic kidney disease, neurodegenerative disease, dementia, functional dependency status, and higher Charlson comorbidity [117].

Conditions related to COPD among which is the declining years, exacerbated inflammatory responses, and diminished muco-ciliary function to make a patient susceptible to viral pulmonary afflictions and virus-triggered exacerbations [118]. It was anticipated that infected patients who smoked tobacco would experience a higher rate of morbidity and death. However, there appears to be a more ambiguous correlation involved.

The elevated inflammatory responses linked with COPD may elucidate the enhanced probability of severe forms of infections in COPD individuals, which may intensify the infection-related cytokine storm [118, 119]. Furthermore, it has been shown that smokers and COPD patients exhibit higher levels of ACE2, which may aid in understanding why the novel coronavirus is more virulent in this patient group [120]. It has been postulated that nicotine's anti-inflammatory qualities may provide protection against COVID-19 complications [121]. The a7-nicotinic acetylcholine receptors (a7nAChR) found on macrophages can be activated by nicotine. By inhibiting the action of nuclear factor kappa B (NF-*κ*B), the a7nAChR receptor's activation reduces the production of inflammatory molecules associated with the cytokine storm [121]. More research must be conducted before any decision can be made about the exact impact of smoking and COPD on COVID-19, even if the reason for the lower-than-anticipated prevalence estimates of COPD in the setting of SARS-CoV-2 infections is still unknown.

Alqahtani et al. concluded in a systematic evaluation that COPD was linked with an enhanced probability of mortality and critical complications associated with the novel coronavirus infection [122]. According to Wang et al.'s meta-analysis of 1,558 infected patients, those who have COPD are significantly more likely to experience COVID-19 worsening compared to those without COPD [123].

It is considered that inhaled corticotherapy in COPD patients does not represent a potential vulnerability to the viral disease but does constitute a possible danger to pneumonia [124].

**Figure 4:**
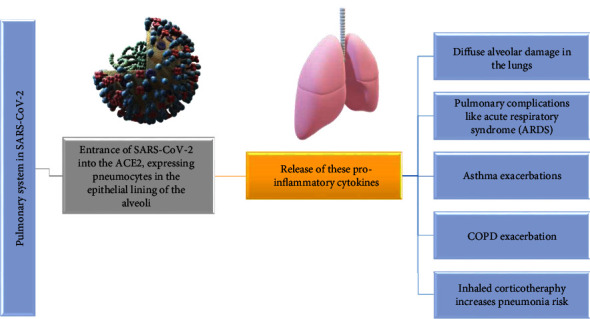
Relevant aspects of the viral infection in respiratory system.

Relevant elements of the viral infection in the respiratory system are highlighted in Figure 4.

### 3.7. Gastro-Intestinal Diseases

Due to COVID-19, patients presenting chronic gastrointestinal (GI) disease could be more susceptible to developing a serious illness. Emerging studies indicate that SARS-CoV-2 has extra-pulmonary manifestations and severe complications, consequently resulting in the failure of multiple organs and death. A limited number of studies indicate that digestive symptoms could predict respiratory issues [5, 125, 126].

Different strategies have been proposed for the GI issues connected to COVID-19. The GI tract has a high prevalence of ACE2 receptors, and SARS-CoV-2 nucleocapsid peptide was found in GI epithelial cells with viral particles isolated from feces, suggesting a direct injury to the GI tract [127]. ACE_2_ has an essential role in intestinal movement and GI inflammation. As a result, when the virus binds to this molecule, it prevents ACE2-expressing enterocytes from conducting their absorptive activities. Furthermore, this process causes malabsorption, aberrant gastrointestinal secretion, and a disturbed enteric nervous system, all of which contribute to diarrhea [128]. Additionally, gut flora might be unbalanced, leading to GI symptoms [129].

The GI system is connected to the respiratory system via the so-called “gut-lung axis.” Strangely enough disturbed gut flora can influence pulmonary functions, and the pulmonary flora can influence the GI tract [130]. On the other hand, antibiotics and corticosteroids, used extensively in medical services that treat patients with COVID-19, lead to dysbiosis and secondary, opening the gate for a multitude of cascading effects (fungal infections, *Clostridioides difficile* infection, inflammatory bowel disease), because they disrupt the functionality of the immunological mechanisms already affected by the viral infection [131–134]. All these processes translate to an increase in mortality, often not related to SARS-CoV-2 infection.

Lastly, pulmonary failure causes prolonged hypoxia which can cause injury and necrosis to GI mucosal cells, leading to ulceration and hemorrhage [127].

Hepatic impairment presents as a common characteristic among COVID-19 patients [135]. Laboratory reports of COVID-19 patients often exhibit enhanced levels of transaminases and hypoalbuminemia, regarded as diagnostic biomarkers [136]. While multiple studies have found a link between liver dysfunction and SARS-CoV-2 infection, it is yet unclear if these anomalies are a direct consequence of the illness. Postmortal examinations revealed that hepatocytes had viral protein in high quantities [137]. The fact that the liver contains a considerable level of ACE2, especially on the membrane of liver endothelial cells, may assist in explaining the results of the examinations [138]. In comparison to liver cells, the bile duct has a more distinct expression of ACE2, with a level of expression similar to that found in alveolar type-2 cells located in the pulmonary system. This demonstrates that COVID-19 is primarily responsible for liver damage by injuring biliary ducts as opposed to hepatocytes [139].

In COVID-19 patients, respiratory failure could induce hepatic injury since the triggered anoxia can eventually impair hepatic function by developing hypoxic hepatitis [140]. Laboratory findings showed that the amount of increased cytokines was noticeably higher in individuals with hepatic impairments compared to those without hepatic damage, indicating that the strong inflammatory response and cytokine storm also contribute to hepatic dysfunction [141]. Therefore, one should use caution when taking into consideration additional aspects that emerge, such as the use of hepatotoxic agents (antibiotics, antivirals, steroids) for the therapeutic management of infected patients, which, according to their own effects, can cause liver damage. Moreover, as infected patients have increased replication of the hepatitis virus, the patient's background of liver infections and diseases is a significant factor that may lead to impaired liver function [142].

Thus far, there is still little explanation regarding the SARS-CoV-2 complex mechanism involving hepatic injury. It is essential to implement prophylactic steps to protect individuals from impairments in their hepatic function considering the accumulating evidence that the novel coronavirus causes hepatic malfunction [143]. Patients infected with the novel coronavirus are recommended to undergo hepatitis C and B screenings, as well as an examination for any additional liver disorders that may already be present [144].

Furthermore, because there is no unambiguous understanding of whether the described hepatic lesions are induced by the novel coronavirus or are caused by the hepatotoxic used agents, it is critical to scrutinize the safety of the effectuated therapeutic protocols [145]. It is also suggested that antiviral therapy be initiated immediately in individuals with liver disorders [146]. Also, it is always advantageous to encourage the incorporation of liver-preserving agents (ammonium glycyrrhizinate) in the treatment that can also reduce the inflammatory process and augment the rate of recovery in infected individuals [147].

Infected patients that present with an associated diarrhea syndrome are prone to critical forms of the illness, needing ICU treatment. Diarrheal conditions should raise the possibility of an infection with the novel coronavirus. The most typical symptoms and signs in infected patients include diarrhea, vomiting, anorexia, and abdominal pain, accompanied by intestinal lesions or inflammation. Systemic inflammation results from the loss of the quality of the intestinal barrier and microbes [148].

A viral load of 54% was quantified in the feces of infected patients, based on a study by Sania et al. [148, 149]. It is still being investigated whether the viral load in the stool is contagious [148].

Infection with the novel coronavirus in the gut causes inflammation because of elevated fecal calprotectin concentrations. The amount of fecal calprotectin in individuals with SARS-CoV-2-related diarrhea is helpful to examine. It would be crucial to examine the mode of fecal-oral transmission of the viral infection [148]. Studies on the intestinal microbiome and the therapeutic purpose of fecal microbial transplantation from healthy donors may be considered in the case of severe forms of infection with the novel coronavirus [148].

In regard to protein S's affinity for the angiotensin II conversion enzyme receptors, SARS-CoV-2 also uses serine 2 transmembrane protease receptors, which are also found in small intestinal epithelial cells [148]. The changes in ACE_2_ might be induced by the novel coronavirus, which may result in intestinal inflammation and diarrhea. Disruption of the intestinal microbiota can be caused by microbes and cytokines, which can cause inflammation and release intestinal cytokines. With age, the intestinal flora is no longer as varied, and the intestinal microbiota is disturbed, leading to a reduction in the ratio of firmicutes to bacteroidetes [148].

The increased death rate among the elderly would be due to low immunity and reduced gut microbiome variety. Zinc and vitamin A deficiency increase the risk of infection [148]. The anti-inflammatory effects of the diet are achieved by elevated-quality proteins, vitamins E and C, carotenoids, polyphenols, and omega-3 fatty acids. The homeostasis of the intestinal microbiota consists of *Prevotella* spp.*, Faecalibacterium* spp., *Bifidobacterium* spp., and *Ruminococcus* spp., which are associated with a reduced level of systemic inflammation. The risk of bacterial or viral inflammation could be reduced by the intake of prebiotics and probiotics [148].

Immunosuppressive medication is administered to individuals suffering from inflammatory bowel disease (IBD). It is known that this therapy is responsible for decreasing the body's response to microorganisms, resulting in these patients being more susceptible to infections. The higher vulnerability to this viral infection in this group of individuals is not yet sufficiently supported by research. According to research, IBD patients have mortality rates and a risk of acquiring severe forms that are similar to those of the general population for the same age range [150].

In addition to antivirals and other therapies, treatment for SARS-CoV-2 also included broad-spectrum antibiotic therapy, used for superimposed bacterial infections. Age, use of antibiotics, hospitalizations, and comorbidities, along with dysbiosis, are elements that influence the emergence of *Clostridioides difficile* infection. The novel coronavirus infection can cause intestinal dysbiosis. Angiotensin II converting enzyme found in the intestine causes gastroenterocolitis-type digestive symptoms in Covid-19 [151]. *Clostridioides difficile* may recur, and fecal microbial transplant therapy would be helpful in this regard. The novel coronavirus can be identified in feces, which is uncertain if COVID-19 can be transmitted by microbial transplantation. *Clostridioides difficile* can remain in the colon, and patients can have a positive result [151].

**Figure 5:**
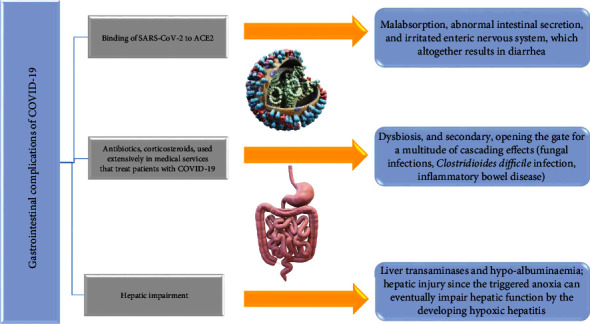
Gastrointestinal complications related to the viral infection.

Figure 5 reveals gastrointestinal complications in COVID-19.

### 3.8. Neurological Diseases

COVID-19 patients can develop a variety of acute neurological disorders, including ageusia and anosmia, epileptiform abnormalities, encephalopathy, cerebral infarction, peripheral neuropathy, and myositis. Long-term neurological syndromes are still not well defined, even though syndromes including dysautonomia, neurocognitive dysfunctions, varied pain syndromes, fatigue, and marked exercise intolerance are frequently described [152].

Of the 214 infected patients hospitalized, 36.4% exhibited neurological damage, according to a retrospective observational case series from Wuhan. This raises the possibility that neurological involvement is an underestimated and underrepresented aspect of the disease's progression. They discovered that neuralgia and vertigo were highly prevalent symptoms among patients with CNS manifestations, with 13% and 17%, respectively. More serious neurological consequences, such as convulsions and strokes, have been uncommon, occurring in only 3% and 0.5% of cases, respectively [153].

They discovered that the majority of neurological symptoms occurred in the early stages of the disease, which could be a critical initial bioindicator of future clinical worsening. Confusion and headache have both been issues indicated by certain patients at the moment of treatment, according to Chen et al.'s retrospective research. However, compared to dyspnea and cough, which are both frequent pulmonary symptoms, neurological dysfunctions were much less prevalent. In a study on 221 individuals, Li et al. found that SARS-CoV-2 causes more serious and potentially long-term neurological problems, and 6% experienced severe neurological illness, including ischemic stroke, bleeding from cerebral vein thrombosis, and death as consequences [137, 154].

Oxley et al. observed five incidences of big vascular stroke in infected individuals less than 50 years old. The two youngest patients, 33 and 37, respectively, seemed to have no prior health records or factors of vulnerability. Two additional investigations evaluated the frequency of thromboembolic incidents in infected patients and found that the rate of ischemic stroke was 1.6% and 2.5%, respectively. It was acknowledged by both studies that the rate of thrombotic problems in those respective hospitals was unusually high. There are undoubtedly multiple factors that increase a patient's probability of developing a thromboembolic stroke in addition to the usual metabolic and cardiac comorbidities, including those linked to a prolonged stay in ICUs [155–157].

Infected patients have a significantly greater frequency of stroke than other disease groups, with higher National Institute of Health Stroke scale (NIHSS) ratings than non-COVID-19 linked stroke patients. Cryptogenic strokes account for more than half of all strokes in COVID-19 individuals, with a larger proportion of major vessel occlusions. Other investigations have found that posterior circulation strokes occur at higher rates than expected (35.3%) [158, 159].

COVID-19-related strokes, which comprise venous and arterial thromboembolic incidents, have been linked to hypercoagulability caused by systemic and focal inflammation [160].

In infected patients, cerebral venous sinus thrombosis (CVST) might be accompanied by an atypically long activated prothromboplastin time (aPTT) and elevated D-dimer values [159, 160].

The expected in-hospital death rate for COVID-19 related CVST in a group of non-ventilated subjects is 40% [161].

Concerns have been raised following observations of vaccine-induced thrombotic thrombocytopenia (VITT) occurring after the administration of COVID-19 vaccines using an adenovirus vector. As of April 4, 2021, 169 incidents of VITT-CVST had been registered with the European Medicines Agency out of 34 million doses of the ChAdOx1 nCoV-19 (i.e., AstraZeneca) vaccine provided, with a frequency of VITT estimated at 1 per 100 000 doses. The rate of VITT-CVST was 0.87 events per million following the administration of 6.86 million units of the Ad26.COV2.S vaccine, implying an adenoviral vector (i.e., Johnson & Johnson/Janssen) [162–166].

There have also been 11 confirmed documented cases and one potential case study of Guillain-Barre syndrome (GBS), as a serious neurological complication of the infection with the novel coronavirus. The 11 patients showed significant differences in the development of GBS symptoms and the typical pulmonary SARS-CoV-2 symptoms. One research described GBS symptoms in a patient who presented to healthcare with only a slight fever, but another paper described nine patients with GBS symptoms five to eleven days after receiving a SARS-CoV2 diagnosis [167–171].

The initial peripheral nervous system symptoms documented in COVID-19 were anosmia and ageusia, and they initiated the importance of understanding SARS-CoV-2's affinity for neural tissues [172]. 5–35% of hospitalized patients described anosmia as a symptom, but this symptom might be higher amid COVID-19 patients who were never hospitalized [173].

The severity of the condition is correlated with EEG anomalies in encephalopathy caused by the infection with the novel coronavirus. The most common manifestation is frontal lobe dysfunction, which has been suggested as an indicator of COVID-19-related encephalopathy [174].

Various COVID-19 patients experience kidney failure, hypoxia, electrolyte imbalances, sedative drugs, and pre-existing comorbidities, as well as changes in mental state (i.e., apathy, disorientation, and coma). Encephalopathy is often present in around a third of patients in critical care, likewise at the beginning of the disease or during the hospitalization, and is associated with poor prognosis together with an increased number of deaths. Numerous reports have claimed that patients infected with the novel coronavirus of all age groups can present themselves with encephalopathy. However, with a severe pulmonary infection, patients around the age of 60 and individuals who have antecedent neurologic conditions, namely stroke, senile dementia, or Parkinson's, are more prone to develop encephalitis [175–178].

**Figure 6:**
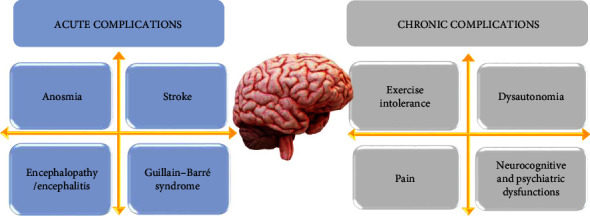
Acute/chronic complications in neurological disease related to COVID-19.

Acute and chronic consequences of COVID-19-related neurological diseases are described in Figure 6.

### 3.9. Autoimmune Disorders

The existence of autoantibodies and recurrent pro-inflammatory processes brought on by impaired immunological tolerance and a dysfunctional immune system harms and impairs the targeted organs [179]. The viral infection also results in this type of immune-mediated effect.

It is expected that individuals with systemic autoimmune disorders will have a greater risk of severe COVID-19 disease and its concomitant complications [180]. By and large, individuals with autoimmune disorders are more susceptible to severe infections. Numerous variables, including diminished adaptive and innate immunological responses and ongoing utilization of immunomodulatory drugs, contribute to this situation, which places the chance of severe infections in these patients at upwards of 2 times more compared to the general population [181]. Recent data indicates that SARS-CoV-2 can precipitate autoimmune diseases and/or auto-inflammatory mechanisms [182].

Based on serological, radiological, and histo-morphological results from 22 patients with COVID-19 admitted in Bundeswehrkrankenhaus Ulm, Germany, during March and April 2020. Gagiannis et al. reviewed the potential implications of autoimmune responses in COVID-19 related respiratory failure. Investigators stated that infection with the novel coronavirus might potentially induce an organ-specific autoimmune reaction in those who are vulnerable to it. [183]. Several autoimmune complications following COVID-19 have been reported, for instance, autoimmune hemolytic anemia, idiopathic thrombocytopenic purpura, and symptoms of Guillain-Barre syndrome [167, 184, 185].

In 33 successive infected patients, research by Pascolini et al. confirmed the presence of anti-antiphospholipid antibodies (APL), anticytoplasmic neutrophil antibodies (ANCA), and antinuclear antibodies (ANA). Researchers found approximately 45% of the subjects had at least one positive autoantibody and were expected to have worse prognoses and markedly increased respiratory rates at admission [186].

A complex chronic autoimmune inflammatory illness called systemic lupus erythematosus (SLE) injures various organs, including skin, kidneys, the heart, and lungs. Patients with SLE are a more special group when it comes to infection with the novel coronavirus. The use of corticosteroids and immunosuppressants, along with the inherent damage to the organs because of lupus, is a factor favoring infection with SARS-CoV-2 in its severe form [187]. Viral infections such as cytomegalovirus, varicella-zoster virus, and upper respiratory tract infections are most common in SLE. The main factor favoring death and hospitalization in patients with SLE is respiratory infections [187]. Epstein–Barr virus is thought to be involved in the pathogenesis of SLE [187, 188]. A critical element in the development of the viral disease is represented by IFN type I, which also has an antiviral role. Individuals with aggressive forms of the viral disease and an enhanced SARS-CoV-2 viral load have an absence of IFN-*β*, low IFN-*α* activity, high TNF and IL-6, and high viral load [187]. In SLE, the complement system is disrupted, with the decrease in the complement level representing the effect of the viral infection. It has been demonstrated that COVID-19 aggravation increases C5a plasma levels in direct proportion. Leptin and alternative complement pathways are thought to be activated by COVID-19 [188].

Destructive thyroiditis leading to thyrotoxicosis is typical in infected patients. Increased concentrations of IL-6 are linked to the preponderance of thyrotoxicosis, and thyroid lesions are secondary to inflammatory thyroiditis [189]. A study presented in 2020 by Muller et al. reports that individuals with aggressive forms of infection in the ICU have a higher frequency of thyrotoxicosis than in patients with mild forms or those with severe lung disease who do not have COVID-19, the frequency is lower [189, 190]. These individuals are mostly male and have an age range of 65 ± 12 years. Risk factors for destructive thyroiditis are enhanced concentrations of TNF-*α* and IL-6 [189]. One of the most typical issues in these patients was fibrillation and thromboembolism. In paraclinical settings, there are high values of C-reactive protein, increased leukocytes with neutrophils, low TSH values, high T3 and T4 values that lead to thyrotoxicosis, positive anti-thyroglobulin antibodies in a small number of events, and negative anti-thyroid peroxidase antibodies [189].

The increased activity of Th1 and Th17 due to COVID-19, secondary to the inflammatory reaction, can lead to autoimmune diseases. Autoimmune abnormalities may include Guillain-Barre syndrome, autoimmune hemolytic anemia, antiphospholipid syndrome, autoimmune thrombocytopenia, and Hashimoto's thyroiditis. Hashimoto's thyroiditis occurs in a few cases after a coronavirus infection [189]. Patients with hypothyroidism who are treated with levothyroxine are not highly susceptible to infection with the novel coronavirus. Moreover, they are not a risk group for hospitalization, which necessitates continued treatment [189].

In patients with IBD, the potential sign of acquiring major viral infections due to immunosuppression that lowers humoral immunity and the action of neutrophils and immunomodulatory and biological treatment is heightened [150]. In a study by Wisniewski et al., IBD patients are three times more likely to develop viral diseases, including viruses like herpes simplex, Epstein–Barr, varicella-zoster, and cytomegalovirus [150, 191]. Patients with IBD due to treatment with antitumor necrosis factor (anti-TNF) are twice as likely to develop opportunistic infections [150, 192]. According to the evidence so far, IBD patients do not present a significantly higher risk of contracting COVID-19 than the overall population [150, 193–195].

About the therapy with azathioprine, methotrexate, anti-TNF, and tofacitinib, the International Organization for the Study of IBD (IOIBD) is doubtful if it would pose a significant risk for the viral infection [150, 193]. Corticosteroids, along with IBD, related disorders, and advanced age, are risk factors for the emergence of virulent SARS-CoV-2 strains [191, 196]. IBD infected patients who have still not developed COVID-19 illness require adjustments in drug therapy such as a 20 mg dose of prednisone to be reduced or replaced with budesonide. Treatment with methotrexate, thiopurine, and tofacitinib should be stopped temporarily, and monoclonal antibody treatment should be postponed by two weeks. [150, 193, 195]. Discontinuation of treatment with thiopurine, methotrexate, and tofacitinib is advised for IBD patients who developed COVID-19 illness and then resumed therapy after the acute phase.

The American Gastroenterology Association indicates that IBD therapy should be re-administered after the symptoms of COVID-19 have subsided or tested negative for COVID-19 [150, 195]. IOIBD guidelines for resuming IBD therapy are to monitor the symptoms rather than testing for COVID-19 [150, 193]. Furthermore, in patients with both IBD and infection with the novel coronavirus, immunosuppressive treatment can be administered after three days of being afebrile and remission of symptoms. Ten days following the positive rapid test result, immunosuppressive treatment may be given to individuals with asymptomatic viral disease [150].

Individuals presenting with multiple sclerosis (MS) are more susceptible to contracting the novel coronavirus since MS is a chronic, inflammatory disease. Due to the associated etiology and pharmacotherapeutic management, MS patients have a higher vulnerability to the viral infection than the overall population.

Federico et al.'s research in Spain (La Rioja) noted that out of 330 patients with MS, 12 contracted SARS-CoV-2 infection, predominantly women, aged 47.91 years, 75% with a form of recessive remission and 25% with progressive secondary [197]. The predominant symptoms among MS patients were cough, fever, dysphagia, myalgias, headache, shortness of breath, and loss of smell. MS patients were admitted at a rate of 22% compared to 36.3% for the overall population [197]. Only one male death was reported at age 74 with progressive secondary MS and no treatment.

MS patients are more inclined to contract SARS-CoV-2 infection and require hospitalization. Moreover, according to the study's findings, MS patients are twice as susceptible to getting SARS-CoV-2 infection as individuals without the condition [197]. The cell-mediated immune responses of MS patients are correlated with a disease-modifying therapy (DMT). Patients not receiving DMT predisposed to COVID-19 are like the general population. Treatment with glatiramer acetate, interferon-beta, and teriflunomide has a reduced predisposition to viral infections because they have a reduced risk of immunosuppression. Teriflunomide and interferon alpha have the property of inhibiting viral replication and having an antiviral effect [198].

Lymphopenia may be due to dimethyl fumarate, especially in early treatment, which increases the potential threat of COVID-19 in lymphopenic individuals [198]. Natalizumab is a highly effective drug deemed with minimal risk of acquiring or exhibiting severe forms of SARS-CoV-2 because it does not interact with lymphocytes. Fingolimod, siponimod, ozanimod (Sphingosine 1-phosphate receptor modulator), ocrelizumab, and rituximab (anti-CD-20 modulators) are drugs with a reduced immunosuppressive effect and a reduced predisposition to infection with the novel coronavirus [198].

The most effective options for the therapeutic management of MS present the highest predisposition to infection due to their action on lymphocytes, being labeled as the most predisposing treatment for this viral infection, affecting immunity, resulting in recurrences or low defense mechanisms against infections [198]. Concerning therapy in individuals with both mild MS and infection with the novel coronavirus, first-line treatment (glatiramer, interferons, dimethyl fumarate, teriflunomide) is preferred. Natalizumab, alemtuzumab, cladribine, or ocrelizumab are indicated in patients with active MS and John Cunningham virus (JCV) positive antibodies [198].

Numerous scientists agree that this viral infection could operate as a major stimulator of inflammatory and immunologic conditions by molecular mimicry, even though the exact mechanisms underpinning the emergence of immune and inflammatory illnesses have not yet been identified [199]. Furthermore, the proinflammatory state and uncontrolled immunological response caused by COVID-19 might prompt and facilitate additional environmental factors in individuals who are inclined to give rise to detected pathologies [199].

### 3.10. Skin Diseases

As a result of COVID-19's mutant nature, Casas et al.'s research study led to the conclusion that the skin lesions can be classified as acral zones of skin redness containing pustules or vesicles (Pseudo-chilblain), numerous different vesicular eruptions, livedo reticularis or necrosis, pruritic lesions, and maculo-papular eruptions. In the initial phase of the disease, vesicular eruptions occur. While the rest come up with other COVID-19 manifestations, the pseudo-chilblain pattern comes late in the course of the viral disease. The extent of COVID-19 progression determines the type and size of the acral lesions [200].

Several of the most common dermatological symptoms in patients infected with the novel coronavirus are acral sores, purpuric rash, maculopapular-erythematous itchiness, livedoid sores, pruritic rash, and vesicular eruptions. These observations could be the first indications of the viral infection. Available treatments for skin disorders include steroids, anticoagulants, and histamine antagonists. When patients develop skin conditions connected to respiratory distress or when they are asymptomatic, the viral disease can be detected using the dermatologic markers of SARS-CoV-2. Furthermore, identifying the dermatologic lesions linked with COVID-19 may aid in the development of a tailored treatment regimen [201].

Acral sores, also known as pernio-like lesions or pseudo-chilblains, are probably the most common type of rash to be documented. They typically affect young adults and develop following the onset of ordinary COVID-19 manifestations [202].

An evidence-based review done by Daneshgaran et al. concluded that the second-highest cutaneous manifestation was erythematous maculo-papular erythema, which usually affected people between the ages of 40 and 60 and appeared simultaneously with other symptoms. Adjacent to regular dermatologic findings were also vesicular rashes and urticarial rashes. Both sorts of rash were present in the adult population. However, vesicular rashes usually appeared after the beginning of integumentary COVID-19 symptoms, whereas urticarial rashes appeared concomitantly. Considering urticarial and erythematous maculo-papular lesions are frequently medication related, their utility to suffice as a COVID-19 distinctive clinical indicator is limited. In contrast, vesicular lesions are more commonly associated with viral exanthems. Vesicular lesions, as a result, could be a more relevant COVID-19 clinical distinguishing feature. COVID-19 investigations, however, have revealed a wide range of vesicular lesions distributions, ranging from diffuse polymorphism to localized monomorphism. More research into the type of vesicular lesion most closely linked to COVID-19 is needed before the diagnostic accuracy of this specific dermatological lesion can be established [203].

Besides, COVID-19 infection, it can also cause coagulopathy and thrombocytopenia, which are both common consequences [204].

Yan et al. conducted retrospective research to examine the relationship between clinical coagulation and acro-ischemic sores attributes in infected patients with severe manifestations. Among additional symptoms that have been admitted to the ICU were finger and toe cyanosis, disseminated intravascular coagulation (DIC), and dry gangrene [205].

A research group led by Piccolo V attempted to compile 63 instances via an online database; subsequently, 54 images were evaluated, out of which erythematous-edematous lesions were found in 31 patients, and blistering laceration in 23 patients [202].

In a study of 132 patients, Fernandez-Nieto et al. described two separate clinical presentations of acute acro-ischemic sores: erythema multiforme-like sores and chilblain-like sores, each with distal localisation [206].

According to a recent Italian study, papulo-vesicular eruptions (varicella-like) are uncommon but distinct fromCOVID-19. The identical clinical manifestations were observed in twenty-two patients, with itching evident in 40.9% of the patients, and the eruption was mostly restricted to the trunk [207].

It is commonly established that infections, especially viral ones, can produce urticarial sores. A potential significant contributor in this direction might be the infection with the novel coronavirus. COVID-19-related urticarial symptoms have been frequently documented in patients who had no previous history of the disease. A rise in the amount of urticaria events detected throughout the pandemic was reported in private clinic research, but no assessment of the presence of COVID-19 in these individuals was attempted. Angioedema is uncommon, and the lesions are disseminated and unrecognizable from other etiology clinically. Urticarial lesions, on the other hand, usually develop in conjunction with other symptoms. With no age demographic predilection, urticaria is linked to moderate systemic conditions with a low death rate. There have been claims that these lesions tend to resolve clinically within a week or with the use of oral antihistamines or in conjunction with oral low-dose systemic steroids [208].

Considering vesico-bullous eruptions are uncommon in viral exanthem and drug-induced reactions, they are considered a distinct characteristic of skin lesions related to COVID-19, which has been used as the main differential diagnosis. These eruptions consist of localized or disseminated vesicles or bullae, which can manifest themselves in the palmoplantar region while sparing the mucous membranes. The onset of these eruptions is almost 2 to 3 days after the typical symptoms and persists for 1 to 2 weeks. Repeatedly, the viral agent was attempted to be isolated from the bullous fluid using the PCR technique, but it was futile. Other viral infections, particularly herpetic ones, must be ruled out, as a case of disseminated herpes with pneumonia has previously been described in a COVID-19 patient, demonstrating the relevance of the clinical investigations [207].

The antecubital fossa and the forearms, dorsal hands and feet, torso, and legs are potential targets for livedoid sores. Rashes typically appear synchronously with other viral disease manifestations and last for a median of approximately 9 days. Furthermore, the most noticeable one, livedo reticularis, can suddenly appear at any stage of the infection, but when it progresses to papulo-necrotic cutaneous vasculitis, it may show issues that result in vascular occlusion, correlating to how severe the infectious disease is. These abnormalities are thought to be the structures most closely associated with fatality, especially in older people with other coexisting diseases and those suffering from highly serious types of the disease. Lesions have been connected to clinical signs, including hematuria and possible renal impairment. Among the highest death rates for infected patients are associated with purpuric and livedoid lesions, which constitute about 10% of COVID-19 patients [209].

### 3.11. Dental Disorders and Issues

Throughout the publications, there have been reports of a plethora of quite diverse oral symptoms linked to COVID-19 patients which comprise erythematous surfaces, reddish macules, petechiae, herpes simplex, candidiasis, hemorrhagic or necrotic ulcerations, white hairy tongue, and pustular enanthema. Due to the lack of comprehensive knowledge, it is still ambiguous if these oral findings are an immediate result of the infection with the novel coronavirus or the adverse events of several medications utilized to treat the viral disease [210].

Prior to the vaccine availability, healthcare systems around the globe encountered a significant challenge because of the rapid expansion of the novel coronavirus. Since the enforced public health measure, a majority of patients have been rendered service less, with consultations being postponed or completely canceled, leading to further complications [211].

Dental professionals and maxillofacial surgeons are at an increased risk of infection because of the sheer close interaction between them and the asymptomatic infected patients who have elevated viral loads in the oral and nasal cavity. During this pandemic, treating patients must be done with extreme caution in order to prevent further transmission of infection.

When the epidemic initially occurred, dental clinics were closed for a predetermined amount of time, and only medical emergencies in dentistry that needed immediate action were carried out [212].

According to a study, online surveys and over-the-phone surveys were carried out aiming to analyze and assess the effects of the virus on dental care. Data was extracted from ten distinct dental practices in total. Approximately 90% of respondents claimed the epidemic has impacted their patient flow, owing to restrictions and prohibitions. And 60% of respondents believe the cost of personal protective equipment (PPE) has grown since the pandemic began, while 40% disagree [213].

Another study by Mac Giolla Phadraig et al. in Ireland using an online questionnaire of dental professionals found that 436 participants indicated a decrease in the proportion of dentists who treat individuals with all types of impairments from before to following the lockdown precautions were implemented. Prior to the lockdown, 22% of respondents reported having no access to any medication; during the lockdown, that number rose to 61%; and after the lockdown, it fell to 44%. Moreover, teledentistry has increased as a result of this. During the initial COVID-19 shutdown, there was a significant negative impact on the provision of dental care to disabled individuals. For those with disabilities, oral healthcare access was severely limited, with sedation and general anesthetic being particularly limited. As new and current services emerge postpandemic, there is a greater need to guarantee that no one is rendered service less [214].

Researchers reached the conclusion that periodontitis was unquestionably associated with an elevated risk of COVID-19 abnormalities, as well as admission to an ICU, the need for assisted ventilation, and mortality, as well as higher blood levels of markers linked to a poor COVID-19 output, especially d-dimer, white blood cells, and CRP [215].

Through cleaving the S-protein, periodontopathic pathogens may play a key role in SARS-CoV-2 penetration, and the molecules produced throughout periodontitis may be implicated in the cytokine storm seen throughout severe symptoms of the viral disease, according to a scoping review of the scientific literature on the correlation between periodontal disorders and the viral disease. Finally, it seems that controlling periodontitis, particularly reducing periodontopathic bacteria as well as local and systemic inflammation, might be a key strategy to inhibit the development of severe COVID-19 forms [216].

ACE_2_ expression, inflammatory markers, and aspiration pneumonia can all be reduced with good dental hygiene and periodontal disease treatment. Therefore, maintaining periodontal general health may reduce the host's susceptibility to the novel coronavirus and assist those who are infected in preventing COVID-19 worsening. Serious conditions like diabetes and COPD benefit from periodontal disease treatment as well [217, 218].

A serious COVID-19 primary infection and associated treatment strategies may ultimately contribute to poor dental health objectives like opportunistic infections, ulcerations, gingivitis, and xerostomia because of a compromised defense mechanism and/or vulnerable oral mucosal surfaces. It is important to understand that a cytokine storm set on by dysfunctions along cellular and humoral routes can worsen autoimmune conditions already present in the oropharynx. Individuals with COVID-19 need additional post-acute management to recuperate from both primary and secondary infectious diseases, as well as close monitoring of their oral health, particularly when moving from the hospital to other care settings and residences. No matter their COVID-19 condition, patients with confirmed oral illnesses whose follow-up treatment has been postponed owing to emergency measures can still receive secondary care attributable to the strengthening of pre-existing oral healthcare centers [219].

The dental hygiene of seriously ill inpatients has been shown to deteriorate as a direct consequence of life-saving measures such as external breathing and blood oxygenation, especially in those receiving intensive care. Because of insufficient oral care as assistance is placed on advanced medical care, mouth breathing, external ventilation, and intubation, hyposalivation can quickly deteriorate oral health and lead to serious issues that impact the lower respiratory tract, comparable to aspiration pneumonia [220].

## 4. Conclusion and Future Interventions

A sudden and rapid outbreak of the COVID-19 pandemic triggered enormous figures of death and sickness across the globe. It also disproportionately impacted individuals with chronic medical conditions. Pre-existing conditions like hypertension, obesity, cardiovascular impairments, diabetes mellitus, and respiratory disorders have become substantial dangerous factors in cases of COVID-19 infection. Individuals with autoimmune disease and dyslipidemia were indicated as particular susceptible category. Reorganizing healthcare assistance to deliver urgent and intensive care for massive amounts of subjects suffering from COVID-19 has not only burdened the healthcare system but also jeopardized the dispensation of routine medical care to patients with chronic conditions. The state of lockdown has adversely impacted the metabolic and psychological health of many patients, particularly the ones that have associated psychological/psychiatric health disorders or potential factors leading to these conditions. Further investigation and subsequent studies will help protect the susceptible high-risk groups from upcoming surges of COVID-19.

## References

[1] Cao J., Tu W. J., Cheng W. (2020). Clinical features and short-term outcomes of 102 patients with coronavirus disease 2019 in wuhan, China. *Clinical Infectious Diseases*.

[2] World Health Organization (2022). Director-General’s remarks at the media briefing on 2019-nCoV on 11 February 2020. https://www.who.int/dg/speeches/detail/who-director-general-s-remarks-at-the-media-briefing-on-2019-ncov-on-11-february-2020.

[3] Kamal M., Abo Omirah M., Hussein A., Saeed H. (2021). Assessment and characterisation of post‐COVID‐19 manifestations. *International Journal of Clinical Practice*.

[4] Booth C. M., Matukas L. M., Tomlinson G. A. (2003). Clinical features and short-term outcomes of 144 patients with SARS in the greater toronto area. *JAMA*.

[5] Wang D., Hu B., Hu C. (2020). Clinical characteristics of 138 hospitalized patients with 2019 novel coronavirus–infected pneumonia in wuhan, China. *JAMA*.

[6] Matsuyama R., Nishiura H., Kutsuna S., Hayakawa K., Ohmagari N. (2016). Clinical determinants of the severity of Middle East respiratory syndrome (MERS): a systematic review and meta-analysis. *BMC Public Health*.

[7] Garbati M. A., Fagbo S. F., Fang V. J. (2016). A comparative study of clinical presentation and risk factors for adverse outcome in patients hospitalised with acute respiratory disease due to MERS coronavirus or other causes. *PLoS One*.

[8] Shiley K. T., Nadolski G., Mickus T., Fishman N. O., Lautenbach E. (2010). Differences in the epidemiological characteristics and clinical outcomes of pandemic (H1N1) 2009 influenza, compared with seasonal influenza. *Infection Control & Hospital Epidemiology*.

[9] Behl T., Kaur I., Aleya L. (2022). CD147-spike protein interaction in COVID-19: get the ball rolling with a novel receptor and therapeutic target. *Science of the Total Environment*.

[10] Wynants L., Van Calster B., Collins G. S. (2020). Prediction models for diagnosis and prognosis of covid-19: systematic review and critical appraisal. *BMJ*.

[11] Page M. J., McKenzie J. E., Bossuyt P. M. (2021). The PRISMA 2020 statement: an updated guideline for reporting systematic reviews. *Journal of Clinical Epidemiology*.

[12] Page M. J., McKenzie J. E., Bossuyt P. M. (2021). Updating guidance for reporting systematic reviews: development of the PRISMA 2020 statement. *Journal of Clinical Epidemiology*.

[13] Hales C. M., Carroll M. D., Fryar C. D., Ogden C. L. (2020). Prevalence of Obesity and Severe Obesity Among Adults: United States, 2017-2018. *NCHS Data Brief*.

[14] Lavie C. J., Sanchis-Gomar F., Henry B. M., Lippi G. (2020). COVID-19 and obesity: links and risks. *Expert Review of Endocrinology and Metabolism*.

[15] Lavie C. J., Laddu D., Arena R., Ortega F. B., Alpert M. A., Kushner R. F. (2018). Healthy weight and obesity prevention: JACC health promotion series. *Journal of the American College of Cardiology*.

[16] Deng G., Yin M., Chen X., Zeng F. (2020). Clinical determinants for fatality of 44,672 patients with COVID-19. *Critical Care*.

[17] Centers for disease control and prevention Atlanta USA (2022). COVID-19 information for specific groups of people. https://www.cdc.gov/coronavirus/2019-ncov/need-extra-precautions/index.html.

[18] Petrilli C. M., Jones S. A., Yang J. (2020). Factors associated with hospital admission and critical illness among 5279 people with coronavirus disease 2019 in New York City: prospective cohort study. *BMJ*.

[19] Cai Q., Chen F., Wang T. (2020). Obesity and COVID-19 severity in a designated hospital in Shenzhen, China. *Diabetes Care*.

[20] Dietz W., Santos-Burgoa C. (2020). Obesity and its implications for COVID-19 mortality. *Obesity*.

[21] Zhu X., Yang L., Huang K. (2020). COVID-19 and obesity: epidemiology, pathogenesis and treatment. *Diabetes Metab Syndr Obes*.

[22] Mohammad S., Aziz R., Al Mahri S. (2021). Obesity and COVID-19: what makes obese host so vulnerable?. *Immunity & Ageing*.

[23] Adler B. J., Kaushansky K., Rubin C. T. (2014). Obesity-driven disruption of haematopoiesis and the bone marrow niche. *Nature Reviews Endocrinology*.

[24] van den Berg S. M., Seijkens T. T. P., Kusters P. J. H. (2016). Diet-induced obesity in mice diminishes hematopoietic stem and progenitor cells in the bone marrow. *The FASEB Journal*.

[25] Bornstein S. R., Dalan R., Hopkins D., Mingrone G., Boehm B. O. (2020). Endocrine and metabolic link to coronavirus infection. *Nature Reviews Endocrinology*.

[26] Benna S. l. (2020). Association of high level gene expression of ACE2 in adipose tissue with mortality of COVID-19 infection in obese patients. *Obesity Medicine Journal*.

[27] Aghili S. M. M., Ebrahimpur M., Arjmand B. (2021). Obesity in COVID-19 era, implications for mechanisms, comorbidities, and prognosis: a review and meta-analysis. *International Journal of Obesity*.

[28] Gonçalves T. J. M., Gonçalves S. E. A. B., Guarnieri A. (2021). Association between low zinc levels and severity of acute respiratory distress syndrome by new coronavirus SARS-CoV-2. *Nutrition in Clinical Practice*.

[29] Elham A. S., Azam K., Azam J., Mostafa L., Nasrin B., Marzieh N. (2021). Serum vitamin D, calcium, and zinc levels in patients with COVID-19. *Clinical Nutrition ESPEN*.

[30] Hariyanto T. I., Kurniawan A. (2020). Dyslipidemia is associated with severe coronavirus disease 2019 (COVID-19) infection. *Diabetes & Metabolic Syndrome: Clinical Research Reviews*.

[31] Wang G., Zhang Q., Zhao X. (2020). Low high-density lipoprotein level is correlated with the severity of COVID-19 patients: an observational study. *Lipids in Health and Disease*.

[32] Van Lenten B. J., Wagner A. C., Navab M. (2004). D-4F, an apolipoprotein A-I mimetic peptide, inhibits the inflammatory response induced by influenza A infection of human type II pneumocytes. *Circulation*.

[33] Dai C., Yao X., Keeran K. J. (2012). Apolipoprotein A-I attenuates ovalbumin-induced neutrophilic airway inflammation via a granulocyte colony-stimulating factor-dependent mechanism. *American Journal of Respiratory Cell and Molecular Biology*.

[34] Kingwell B. A., Chapman M. J., Kontush A., Miller N. E. (2014). HDL-targeted therapies: progress, failures and future. *Nature Reviews Drug Discovery*.

[35] van der Stoep M., Korporaal S. J., Van Eck M. (2014). High-density lipoprotein as a modulator of platelet and coagulation responses. *Cardiovascular Research*.

[36] Wu K.-S., Lin P.-C., Chen Y.-S., Pan T.-C., Tang P.-L. (2021). The use of statins was associated with reduced COVID-19 mortality: a systematic review and meta-analysis. *Annals of Medicine*.

[37] Gheorghe G., Toth P. P., Bungau S. (2020). Cardiovascular risk and statin therapy considerations in women. *Diagnostics*.

[38] The L. (2011). The diabetes pandemic. *The Lancet*.

[39] Cortes Garcia M., Sierra Moros M. J., Santa-Olalla Peralta P., Hernandez-Barrera V., Jimenez-Garcia R., Pachon I. (2012). Clinical characteristics and outcomes of diabetic patients who were hospitalised with 2009 pandemic influenza A H1N1 infection. *Journal of Infection*.

[40] Allard R., Leclerc P., Tremblay C., Tannenbaum T.-N. (2010). Diabetes and the severity of pandemic influenza A (H1N1) infection. *Diabetes Care*.

[41] Gentile S., Strollo F., Ceriello A. (2020). COVID-19 infection in Italian people with diabetes: lessons learned for our future (an experience to be used). *Diabetes Research and Clinical Practice*.

[42] Dal Canto E., Ceriello A., Rydén L. (2020). Diabetes as a cardiovascular risk factor: an overview of global trends of macro and micro vascular complications. *European Journal of Preventive Cardiology*.

[43] Muniyappa R., Gubbi S. (2020). COVID-19 pandemic, coronaviruses, and diabetes mellitus. *American Journal of Physiology—Endocrinology And Metabolism*.

[44] Barber T. M. (2020). COVID-19 and diabetes mellitus: implications for prognosis and clinical management. *Expert Review of Endocrinology and Metabolism*.

[45] Klekotka R., Mizgała E., Król W. (2015). The etiology of lower respiratory tract infections in people with diabetes. *Advances in Respiratory Medicine*.

[46] Lim S., Bae J. H., Kwon H.-S., Nauck M. A. (2021). COVID-19 and diabetes mellitus: from pathophysiology to clinical management. *Nature Reviews Endocrinology*.

[47] Ji H.-L., Zhao R., Matalon S., Matthay M. A. (2020). Elevated plasmin(ogen) as a common risk factor for COVID-19 susceptibility. *Physiological Reviews*.

[48] Guo W., Li M., Dong Y. (2020). Diabetes is a risk factor for the progression and prognosis of COVID-19. *Diabetes*.

[49] Seiglie J., Platt J., Cromer S. J. (2020). Diabetes as a risk factor for poor early outcomes in patients hospitalized with COVID-19. *Diabetes Care*.

[50] Mansour A., Sajjadi-Jazi S. M., Kasaeian A. (2020). Clinical characteristics and outcomes of diabetics hospitalized for COVID-19 infection: a single-centered, retrospective, observational study. *EXCLI journal*.

[51] Li J., Wang X., Chen J., Zuo X., Zhang H., Deng A. (2020). COVID-19 infection may cause ketosis and ketoacidosis. *Diabetes, Obesity and Metabolism*.

[52] Unsworth R., Wallace S., Oliver N. S. (2020). New-onset type 1 diabetes in children during COVID-19: multicenter regional findings in the UK. *Diabetes Care*.

[53] Caruso P., Longo M., Signoriello S. (2020). Diabetic foot problems during the COVID-19 pandemic in a tertiary care center: the emergency among the emergencies. *Diabetes Care*.

[54] Cariou B., Hadjadj S., Wargny M. (2020). Phenotypic characteristics and prognosis of inpatients with COVID-19 and diabetes: the CORONADO study. *Diabetologia*.

[55] Wang S., Ma P., Zhang S. (2020). Fasting blood glucose at admission is an independent predictor for 28-day mortality in patients with COVID-19 without previous diagnosis of diabetes: a multi-centre retrospective study. *Diabetologia*.

[56] Singh A. K., Gupta R., Ghosh A., Misra A. (2020). Diabetes in COVID-19: prevalence, pathophysiology, prognosis and practical considerations. *Diabetes & Metabolic Syndrome: Clinical Research Reviews*.

[57] Zhang M., He J.-Q. (2020). Impacts of metformin on tuberculosis incidence and clinical outcomes in patients with diabetes: a systematic review and meta-analysis. *European Journal of Clinical Pharmacology*.

[58] Gorricho J., Garjón J., Alonso A. (2017). Use of oral antidiabetic agents and risk of community-acquired pneumonia: a nested case–control study. *British Journal of Clinical Pharmacology*.

[59] Zhang W., Xu Y.-Z., Liu B. (2014). Pioglitazone upregulates angiotensin converting enzyme 2 expression in insulin-sensitive tissues in rats with high-fat diet-induced nonalcoholic steatohepatitis. *The Scientific World Journal*.

[60] de la Cuesta-Zuluaga J., Mueller N. T., Corrales-Agudelo V. (2017). Metformin is associated with higher relative abundance of mucin-degrading akkermansia muciniphila and several short-chain fatty acid-producing microbiota in the gut. *Diabetes Care*.

[61] Ho T.-W., Huang C.-T., Tsai Y.-J., Lien A. S.-Y., Lai F., Yu C.-J. (2019). Metformin use mitigates the adverse prognostic effect of diabetes mellitus in chronic obstructive pulmonary disease. *Respiratory Research*.

[62] Crouse A. B., Grimes T., Li P., Might M., Ovalle F., Shalev A. (2021). Metformin use is associated with reduced mortality in a diverse population with COVID-19 and diabetes. *Frontiers in Endocrinology*.

[63] Mauvais-Jarvis F. (2020). Aging, male sex, obesity, and metabolic inflammation create the perfect storm for COVID-19. *Diabetes*.

[64] Liu P. P., Blet A., Smyth D., Li H. (2020). The science underlying COVID-19. *Circulation*.

[65] Gheorghe G., Ilie M., Bungau S., Stoian A. M. P., Bacalbasa N., Diaconu C. C. (2021). Is there a relationship between COVID-19 and hyponatremia?. *Medicina-Lithuania*.

[66] Li B., Yang J., Zhao F. (2020). Prevalence and impact of cardiovascular metabolic diseases on COVID-19 in China. *Clinical Research in Cardiology*.

[67] Zhonghua L., Xing B., Zhi Z. (2020). Novel coronavirus pneumonia emergency response epidemiology team. *The epidemiological characteristics of an outbreak of 2019*.

[68] Gao C., Cai Y., Zhang K. (2020). Association of hypertension and antihypertensive treatment with COVID-19 mortality: a retrospective observational study. *European Heart Journal*.

[69] Adler A. I., Stratton I. M., Neil H. A. W. (2000). Association of systolic blood pressure with macrovascular and microvascular complications of type 2 diabetes (UKPDS 36): prospective observational study. *BMJ*.

[70] Petrie J. R., Guzik T. J., Touyz R. M. (2018). Diabetes, hypertension, and cardiovascular disease: clinical insights and vascular mechanisms. *Canadian Journal of Cardiology*.

[71] Klimas J., Olvedy M., Ochodnicka-Mackovicova K. (2015). Perinatally administered losartan augments renal ACE2 expression but not cardiac or renal Mas receptor in spontaneously hypertensive rats. *Journal of Cellular and Molecular Medicine*.

[72] Ferrario C. M., Jessup J., Chappell M. C. (2005). Effect of angiotensin-converting enzyme inhibition and angiotensin II receptor blockers on cardiac angiotensin-converting enzyme 2. *Circulation*.

[73] Kuster G. M., Pfister O., Burkard T. (2020). SARS-CoV2: should inhibitors of the renin–angiotensin system be withdrawn in patients with COVID-19?. *European Heart Journal*.

[74] (2020). Clinical characteristics of covid-19 in China. *New England Journal of Medicine*.

[75] Chalmers J. D., Singanayagam A., Hill A. T. (2008). Systolic blood pressure is superior to other haemodynamic predictors of outcome in community acquired pneumonia. *Thorax*.

[76] Lippi G., Wong J., Henry B. M. (2020). Hypertension and its severity or mortality in Coronavirus Disease 2019 (COVID-19): a pooled analysis. *Polish Archives of Internal Medicine*.

[77] Guo J., Wei X., Li Q. (2020). Single-cell RNA analysis on ACE2 expression provides insights into SARS-CoV-2 potential entry into the bloodstream and heart injury. *Journal of Cellular Physiology*.

[78] Siripanthong B., Nazarian S., Muser D. (2020). Recognizing COVID-19–related myocarditis: The possible pathophysiology and proposed guideline for diagnosis and management. *Heart Rhythm*.

[79] Blackburn R., Zhao H., Pebody R., Hayward A., Warren-Gash C. (2018). Laboratory-confirmed respiratory infections as predictors of hospital admission for myocardial infarction and stroke: time-series analysis of English data for 2004–2015. *Clinical Infectious Diseases*.

[80] Guo T., Fan Y., Chen M. (2020). Cardiovascular implications of fatal outcomes of patients with coronavirus disease 2019 (COVID-19). *JAMA Cardiology*.

[81] Gawałko M., Kapłon-Cieślicka A., Hohl M., Dobrev D., Linz D. (2020). COVID-19 associated atrial fibrillation: incidence, putative mechanisms and potential clinical implications. *IJC Heart & Vasculature*.

[82] Zuin M., Rigatelli G., Bilato C., Zanon F., Zuliani G., Roncon L. (2021). Pre-existing atrial fibrillation is associated with increased mortality in COVID-19 Patients. *Journal of Interventional Cardiac Electrophysiology*.

[83] Nishiga M., Wang D. W., Han Y., Lewis D. B., Wu J. C. (2020). COVID-19 and cardiovascular disease: from basic mechanisms to clinical perspectives. *Nature Reviews Cardiology*.

[84] Driggin E., Madhavan M. V., Bikdeli B. (2020). Cardiovascular considerations for patients, health care workers, and health systems during the COVID-19 pandemic. *Journal of the American College of Cardiology*.

[85] Chen T., Wu D., Chen H. (2020). Clinical characteristics of 113 deceased patients with coronavirus disease 2019: retrospective study. *BMJ*.

[86] Yang X., Yu Y., Xu J. (2020). Clinical course and outcomes of critically ill patients with SARS-CoV-2 pneumonia in Wuhan, China: a single-centered, retrospective, observational study. *The Lancet Respiratory Medicine*.

[87] Husain-Syed F., Slutsky A. S., Ronco C. (2016). Lung–kidney cross-talk in the critically ill patient. *American Journal of Respiratory and Critical Care Medicine*.

[88] van den Akker J. P. C., Egal M., Groeneveld A. B. J. (2013). Invasive mechanical ventilation as a risk factor for acute kidney injury in the critically ill: a systematic review and meta-analysis. *Critical Care*.

[89] Głowacka M., Lipka S., Młynarska E., Franczyk B., Rysz J. (2021). Acute kidney injury in COVID-19. *International Journal of Molecular Sciences*.

[90] Martinez-Rojas M. A., Vega-Vega O., Bobadilla N. A. (2020). Is the kidney a target of SARS-CoV-2?. *American Journal of Physiology - Renal Physiology*.

[91] Zahid U., Ramachandran P., Spitalewitz S. (2020). Acute kidney injury in COVID-19 patients: an inner city hospital experience and policy implications. *American Journal of Nephrology*.

[92] Adapa S., Chenna A., Balla M. (2020). COVID-19 pandemic causing acute kidney injury and impact on patients with chronic kidney disease and renal transplantation. *Journal of Clinical Medicine Research*.

[93] Ronco C., Reis T. (2020). Kidney involvement in COVID-19 and rationale for extracorporeal therapies. *Nature Reviews Nephrology*.

[94] Diao B., Wang C., Wang R. (2021). Human kidney is a target for novel severe acute respiratory syndrome coronavirus 2 infection. *Nature Communications*.

[95] Minami T., Iwata Y., Wada T. (2020). Renal complications in coronavirus disease 2019: a systematic review. *Inflammation and Regeneration*.

[96] Nadim M. K., Forni L. G., Mehta R. L. (2020). COVID-19-associated acute kidney injury: consensus report of the 25th Acute Disease Quality Initiative (ADQI) Workgroup. *Nature Reviews Nephrology*.

[97] Henry B. M., Lippi G. (2020). Chronic kidney disease is associated with severe coronavirus disease 2019 (COVID-19) infection. *International Urology and Nephrology*.

[98] Salvadori M., Tsalouchos A. (2020). SARS-CoV-2-Associated Nephropathy: Direct Renal Infection and Damage. https://www.emjreviews.com/nephrology/article/sars-cov-2-associated-nephropathy-direct-renal-infection-and-damage/.

[99] Vincent J.-L., Taccone F. S. (2020). Understanding pathways to death in patients with COVID-19. *The Lancet Respiratory Medicine*.

[100] Zhang H., Penninger J. M., Li Y., Zhong N., Slutsky A. S. (2020). Angiotensin-converting enzyme 2 (ACE2) as a SARS-CoV-2 receptor: molecular mechanisms and potential therapeutic target. *Intensive Care Medicine*.

[101] Chousterman B. G., Swirski F. K., Weber G. F. (2017). Cytokine storm and sepsis disease pathogenesis. *Seminars in Immunopathology*.

[102] Schnappauf O., Chae J. J., Kastner D. L., Aksentijevich I. (2019). The pyrin inflammasome in health and disease. *Frontiers in Immunology*.

[103] Xu Z., Shi L., Wang Y. (2020). Pathological findings of COVID-19 associated with acute respiratory distress syndrome. *The Lancet Respiratory Medicine*.

[104] Abrams E. M., W‘t Jong G., Yang C. L. (2020). Asthma and COVID-19. *Canadian Medical Association Journal*.

[105] Zheng X.-Y., Xu Y.-J., Guan W.-J., Lin L.-F. (2018). Regional, age and respiratory-secretion-specific prevalence of respiratory viruses associated with asthma exacerbation: a literature review. *Archives of Virology*.

[106] Dunican E. M., Fahy J. V. (2015). The role of type 2 inflammation in the pathogenesis of asthma exacerbations. *Annals of the American Thoracic Society*.

[107] Liu S., Zhi Y., Ying S. (2020). COVID-19 and asthma: reflection during the pandemic. *Clinical Reviews in Allergy and Immunology*.

[108] Carli G., Cecchi L., Stebbing J., Parronchi P., Farsi A. Is (2021). Asthma protective against COVID-19?. *Allergy*.

[109] Beurnier A., Jutant E.-M., Jevnikar M. (2020). Characteristics and outcomes of asthmatic patients with COVID-19 pneumonia who require hospitalisation. *European Respiratory Journal*.

[110] Yang J. M., Koh H. Y., Moon S. Y. (2020). Allergic disorders and susceptibility to and severity of COVID-19: A nationwide cohort study. *Journal of Allergy and Clinical Immunology*.

[111] Ferastraoaru D., Hudes G., Jerschow E. (2021). Eosinophilia in asthma patients is protective against severe COVID-19 illness. *Journal of Allergy and Clinical Immunology: In Practice*.

[112] Peters M. C., Sajuthi S., Deford P. (2020). COVID-19–related genes in sputum cells in asthma. Relationship to demographic features and corticosteroids. *American Journal of Respiratory and Critical Care Medicine*.

[113] Shi T., Pan J., Katikireddi S. V. (2022). Risk of COVID-19 hospital admission among children aged 5–17 years with asthma in Scotland: a national incident cohort study. *The Lancet Respiratory Medicine*.

[114] Zhu Z., Hasegawa K., Ma B., Fujiogi M., Camargo C. A., Liang L. (2020). Association of asthma and its genetic predisposition with the risk of severe COVID-19. *The Journal of Allergy and Clinical Immunology*.

[115] Qin C., Zhou L., Hu Z. (2020). Dysregulation of immune response in patients with coronavirus 2019 (COVID-19) in wuhan, China. *Clinical Infectious Diseases*.

[116] Bauer C. M. T., Morissette M. C., Stämpfli M. R. (2013). The influence of cigarette smoking on viral infections: translating bench science to impact COPD pathogenesis and acute exacerbations of COPD clinically. *Chest*.

[117] Antúnez M. G., Míguez A. M., Estrada A. D. B. (2020). Clinical characteristics and prognosis of COPD patients hospitalized with SARS-CoV-2. *International Journal of Chronic Obstructive Pulmonary Disease*.

[118] Lippi G., Henry B. M. (2020). Chronic obstructive pulmonary disease is associated with severe coronavirus disease 2019 (COVID-19). *Respiratory Medicine*.

[119] Restrepo M. I., Sibila O., Anzueto A. (2018). Pneumonia in patients with chronic obstructive pulmonary disease. *Tuberculosis and Respiratory Diseases*.

[120] Leung J. M., Yang C. X., Tam A. (2020). ACE-2 expression in the small airway epithelia of smokers and COPD patients: implications for COVID-19. *European Respiratory Journal*.

[121] Gonzalez-Rubio J., Navarro-Lopez C., Lopez-Najera E. (2020). Cytokine release syndrome (CRS) and nicotine in COVID-19 patients: trying to calm the storm. *Frontiers in Immunology*.

[122] Alqahtani J. S., Oyelade T., Aldhahir A. M. (2020). Prevalence, severity and mortality associated with COPD and smoking in patients with COVID-19: a rapid systematic review and meta-analysis. *PLoS One*.

[123] Wang B., Li R., Lu Z., Huang Y. (2020). Does comorbidity increase the risk of patients with COVID-19: evidence from meta-analysis. *Aging (Albany NY)*.

[124] Edis E. Ç. (2020). Chronic pulmonary diseases and COVID-19. *Turkish Thoracic Journal*.

[125] Han C., Duan C., Zhang S. (2020). Digestive symptoms in COVID-19 patients with mild disease severity: clinical presentation, stool viral RNA testing, and outcomes. *Official journal of the American College of Gastroenterology|ACG*.

[126] Hunt R. H., East J. E., Lanas A. (2021). COVID-19 and gastrointestinal disease: implications for the gastroenterologist. *Digestive Diseases*.

[127] Tian Y., Rong L., Nian W., He Y. (2020). Review article: gastrointestinal features in COVID-19 and the possibility of faecal transmission. *Alimentary Pharmacology & Therapeutics*.

[128] Zhang H., Kang Z., Gong H. (2020). The digestive system is a potential route of 2019-nCov infection: a bioinformatics analysis based on single-cell transcriptomes. *bioRxiv*.

[129] Pan L., Mu M., Yang P. (2020). Clinical characteristics of COVID-19 patients with digestive symptoms in Hubei, China: a descriptive, cross-sectional, multicenter study. *Official journal of the American College of Gastroenterology|ACG*.

[130] Budden K. F., Gellatly S. L., Wood D. L. A. (2017). Emerging pathogenic links between microbiota and the gut–lung axis. *Nature Reviews Microbiology*.

[131] Nishida A., Inoue R., Inatomi O., Bamba S., Naito Y., Andoh A. (2018). Gut microbiota in the pathogenesis of inflammatory bowel disease. *Clinical Journal of Gastroenterology*.

[132] Negrut N., Nistor-Cseppento D. C., Khan S. A. (2020). *Clostridium difficile* infection epidemiology over a period of 8 Years—a single centre study. *Sustainability*.

[133] Negrut N., Bungau S., Behl T. (2020). Risk factors associated with recurrent Clostridioides difficile infection. *Healthcare*.

[134] Negruţ N., Khan S., Bungau S. (2020). Diagnostic challenges in gastrointestinal infections. *Military Medicine*.

[135] Garrido I., Liberal R., Macedo G. (2020). Review article: COVID-19 and liver disease—what we know on 1st May 2020. *Alimentary Pharmacology & Therapeutics*.

[136] Liu W., Tao Z.-W., Wang L. (2020). Analysis of factors associated with disease outcomes in hospitalized patients with 2019 novel coronavirus disease. *Chinese Medical Journal*.

[137] Chen N., Zhou M., Dong X. (2020). Epidemiological and clinical characteristics of 99 cases of 2019 novel coronavirus pneumonia in Wuhan, China: a descriptive study. *The Lancet*.

[138] Hamming I., Timens W., Bulthuis M., Lely A., Navis G., van Goor H. (2004). Tissue distribution of ACE2 protein, the functional receptor for SARS coronavirus. A first step in understanding SARS pathogenesis. *The Journal of Pathology*.

[139] Chai X., Hu L., Zhang Y. (2020). Specific ACE2 expression in cholangiocytes may cause liver damage after 2019-nCoV infection. *bioRxiv*.

[140] Sun J., Aghemo A., Forner A., Valenti L. (2020). COVID-19 and liver disease. *Liver International*.

[141] Jin X., Lian J.-S., Hu J.-H. (2020). Epidemiological, clinical and virological characteristics of 74 cases of coronavirus-infected disease 2019 (COVID-19) with gastrointestinal symptoms. *Gut*.

[142] Mantovani A., Beatrice G., Dalbeni A. (2020). Coronavirus disease 2019 and prevalence of chronic liver disease: a meta-analysis. *Liver International*.

[143] Boettler T., Newsome P. N., Mondelli M. U. (2020). Care of patients with liver disease during the COVID-19 pandemic: EASL-ESCMID position paper. *JHEP Reports*.

[144] Wang X., Lei J., Li Z., Yan L. (2021). Potential effects of coronaviruses on the liver: an update. *Frontiers of Medicine*.

[145] Yu D., Du Q., Yan S. (2021). Liver injury in COVID-19: clinical features and treatment management. *Virology Journal*.

[146] Boettler T., Marjot T., Newsome P. N. (2020). Impact of COVID-19 on the care of patients with liver disease: EASL-ESCMID position paper after 6 months of the pandemic. *JHEP Reports*.

[147] Yu Y.-C., Mao Y.-M., Chen C.-W. (2017). CSH guidelines for the diagnosis and treatment of drug-induced liver injury. *Hepatology International*.

[148] Villapol S. (2020). Gastrointestinal symptoms associated with COVID-19: impact on the gut microbiome. *Translational Research*.

[149] Xie C., Jiang L., Huang G. (2020). Comparison of different samples for 2019 novel coronavirus detection by nucleic acid amplification tests. *International Journal of Infectious Diseases*.

[150] Hashash J. G., Jabak S., Francis F. F., Regueiro M. (2020). Should we Be screening for SARS-CoV-2 in IBD patients before initiation of biologic therapy?. *Inflammatory Bowel Diseases*.

[151] Khanna S., Kraft C. S. (2021). The interplay of SARS-CoV-2 and Clostridioides difficile infection. *Future Microbiology*.

[152] Balcom E. F., Nath A., Power C. (2021). Acute and chronic neurological disorders in COVID-19: potential mechanisms of disease. *Brain*.

[153] Mao L., Jin H., Wang M. (2020). Neurologic manifestations of hospitalized patients with coronavirus disease 2019 in wuhan, China. *JAMA Neurology*.

[154] Li Y., Li M., Wang M. (2020). Acute cerebrovascular disease following COVID-19: a single center, retrospective, observational study. *Stroke Vasc Neurol*.

[155] Oxley T. J., Mocco J., Majidi S. (2020). Large-vessel stroke as a presenting feature of covid-19 in the young. *New England Journal of Medicine*.

[156] Klok F. A., Kruip M., van der Meer N. J. M. (2020). Incidence of thrombotic complications in critically ill ICU patients with COVID-19. *Thrombosis Research*.

[157] Lodigiani C., Iapichino G., Carenzo L. (2020). Venous and arterial thromboembolic complications in COVID-19 patients admitted to an academic hospital in Milan, Italy. *Thrombosis Research*.

[158] Hernández-Fernández F., Sandoval Valencia H., Barbella-Aponte R. A. (2020). Cerebrovascular disease in patients with COVID-19: neuroimaging, histological and clinical description. *Brain*.

[159] Yaghi S., Ishida K., Torres J. (2020). SARS-CoV-2 and stroke in a New York healthcare system. *Stroke*.

[160] Malas M. B., Naazie I. N., Elsayed N., Mathlouthi A., Marmor R., Clary B. (2020). Thromboembolism risk of COVID-19 is high and associated with a higher risk of mortality: a systematic review and meta-analysis. *EClinicalMedicine*.

[161] Baldini T., Asioli G. M., Romoli M. (2021). Cerebral venous thrombosis and severe acute respiratory syndrome coronavirus-2 infection: a systematic review and meta-analysis. *European Journal of Neurology*.

[162] Schultz N. H., Sørvoll I. H., Michelsen A. E. (2021). Thrombosis and thrombocytopenia after ChAdOx1 nCoV-19 vaccination. *New England Journal of Medicine*.

[163] Bayas A., Menacher M., Christ M., Behrens L., Rank A., Naumann M. (2021). Bilateral superior ophthalmic vein thrombosis, ischaemic stroke, and immune thrombocytopenia after ChAdOx1 nCoV-19 vaccination. *Lancet*.

[164] Greinacher A., Thiele T., Warkentin T. E., Weisser K., Kyrle P. A., Eichinger S. (2021). Thrombotic thrombocytopenia after ChAdOx1 nCov-19 vaccination. *New England Journal of Medicine*.

[165] Sadoff J., Davis K., Douoguih M. (2021). Thrombotic thrombocytopenia after Ad26.COV2.S vaccination—response from the manufacturer. *New England Journal of Medicine*.

[166] Muir K.-L., Kallam A., Koepsell S. A., Gundabolu K. (2021). Thrombotic thrombocytopenia after Ad26.COV2.S vaccination. *New England Journal of Medicine*.

[167] Toscano G., Palmerini F., Ravaglia S. (2020). Guillain–barré syndrome associated with SARS-CoV-2. *New England Journal of Medicine*.

[168] Coen M., Jeanson G., Culebras Almeida L. A. (2020). Guillain-Barré syndrome as a complication of SARS-CoV-2 infection. *Brain, Behavior, and Immunity*.

[169] Camdessanche J. P., Morel J., Pozzetto B., Paul S., Tholance Y., Botelho-Nevers E. (2020). COVID-19 may induce Guillain-Barré syndrome. *Revue Neurologique*.

[170] Sedaghat Z., Karimi N. (2020). Guillain Barre syndrome associated with COVID-19 infection: a case report. *Journal of Clinical Neuroscience*.

[171] Virani A., Rabold E., Hanson T. (2020). Guillain-Barré Syndrome associated with SARS-CoV-2 infection. *IDCases*.

[172] Pouga L. (2021). Encephalitic syndrome and anosmia in COVID-19: do these clinical presentations really reflect SARS-CoV-2 neurotropism? A theory based on the review of 25 COVID-19 cases. *Journal of Medical Virology*.

[173] Yan C. H., Faraji F., Prajapati D. P., Ostrander B. T., DeConde A. S. (2020). Self-reported olfactory loss associates with outpatient clinical course in COVID-19. *Int Forum Allergy Rhinol*.

[174] Antony A. R., Haneef Z. (2020). Systematic review of EEG findings in 617 patients diagnosed with COVID-19. *Seizure*.

[175] Toniolo S., Di Lorenzo F., Scarioni M., Frederiksen K. S., Nobili F. (2021). Is the frontal lobe the primary target of SARS-CoV-2?. *J Alzheimers Dis*.

[176] Pensato U., Muccioli L., Pasini E. (2020). Encephalopathy in COVID-19 presenting with acute aphasia mimicking stroke. *Frontiers in Neurology*.

[177] Liotta E. M., Batra A., Clark J. R. (2020). Frequent neurologic manifestations and encephalopathy-associated morbidity in Covid-19 patients. *Ann Clin Transl Neurol*.

[178] Hosp J. A., Dressing A., Blazhenets G. (2021). Cognitive impairment and altered cerebral glucose metabolism in the subacute stage of COVID-19. *Brain*.

[179] Hejrati A., Rafiei A., Soltanshahi M. (2020). Innate immune response in systemic autoimmune diseases: a potential target of therapy. *Inflammopharmacology*.

[180] Figueroa-Parra G., Aguirre-Garcia G. M., Gamboa-Alonso C. M., Camacho-Ortiz A., Galarza-Delgado D. A. (2020). Are my patients with rheumatic diseases at higher risk of COVID-19?. *Annals of the Rheumatic Diseases*.

[181] Listing J., Gerhold K., Zink A. (2012). The risk of infections associated with rheumatoid arthritis, with its comorbidity and treatment. *Rheumatology*.

[182] Caso F., Costa L., Ruscitti P. (2020). Could Sars-coronavirus-2 trigger autoimmune and/or autoinflammatory mechanisms in genetically predisposed subjects?. *Autoimmunity Reviews*.

[183] Gagiannis D., Steinestel J., Hackenbroch C. (2020). Clinical, serological, and histopathological similarities between severe COVID-19 and acute exacerbation of connective tissue disease-associated interstitial lung disease (CTD-ILD). *Frontiers in Immunology*.

[184] Lazarian G., Quinquenel A., Bellal M. (2020). Autoimmune haemolytic anaemia associated with COVID-19 infection. *British Journal of Haematology*.

[185] Zulfiqar A.-A., Lorenzo-Villalba N., Hassler P., Andrès E. (2020). Immune thrombocytopenic purpura in a patient with covid-19. *New England Journal of Medicine*.

[186] Pascolini S., Vannini A., Deleonardi G. (2021). COVID-19 and immunological dysregulation: can autoantibodies be useful?. *Clinical and Translational Science*.

[187] Fernandez-Ruiz R., Paredes J. L., Niewold T. B. (2021). COVID-19 in patients with systemic lupus erythematosus: lessons learned from the inflammatory disease. *Translational Research*.

[188] James J. A., Robertson J. M. (2012). Lupus and epstein-barr. *Current Opinion in Rheumatology*.

[189] Caron P. (2021). Thyroiditis and SARS-CoV-2 pandemic: a review. *Endocrine*.

[190] Muller I., Cannavaro D., Dazzi D. (2020). SARS-CoV-2-related atypical thyroiditis. *Lancet Diabetes & Endocrinology*.

[191] Wisniewski A., Kirchgesner J., Seksik P. (2020). Increased incidence of systemic serious viral infections in patients with inflammatory bowel disease associates with active disease and use of thiopurines. *United European Gastroenterology Journal*.

[192] Ford A. C., Peyrin-Biroulet L. (2013). Opportunistic infections with anti-tumor necrosis factor-*α* therapy in inflammatory bowel disease: meta-analysis of randomized controlled trials. *Official journal of the American College of Gastroenterology | ACG*.

[193] Rubin D. T., Abreu M. T., Rai V. (2020). Management of Patients With Crohn’s Disease and Ulcerative Colitis During the Coronavirus Disease-2019 Pandemic: Results of an International Meeting. *Gastroenterology*.

[194] Gutin L. S., Lam A. Y., Velayos F. S., Santos S. A. (2020). Going viral: management of IBD in the era of the COVID-19 pandemic. *Digestive Diseases and Sciences*.

[195] Rubin D. T., Feuerstein J. D., Wang A. Y., Cohen R. D. (2020). AGA clinical practice update on management of inflammatory bowel disease during the COVID-19 pandemic: expert commentary. *Gastroenterology*.

[196] Brenner E. J., Ungaro R. C., Gearry R. B. (2020). Corticosteroids, but not TNF antagonists, are associated with adverse COVID-19 outcomes in patients with inflammatory bowel diseases: results from an international registry. *Gastroenterology*.

[197] Castillo Álvarez F., López Pérez M. Á., Marzo Sola M. E. (2020). Risk of SARS-CoV-2 infection and clinical outcomes in multiple sclerosis patients in La Rioja (Spain): riesgo de infección por SARS-CoV-2 y resultados clínicos en pacientes con esclerosis múltiple en la Rioja (España). *Medicina Clínica*.

[198] Diaz de la Fe A., Peláez Suárez A. A., Fuentes Campos M. (2021). SARS-CoV-2 infection and risk management in multiple sclerosis. *Diseases*.

[199] Galeotti C., Bayry J. (2020). Autoimmune and inflammatory diseases following COVID-19. *Nature Reviews Rheumatology*.

[200] Galván Casas C., Català A., Carretero Hernández G. (2020). Classification of the cutaneous manifestations of COVID-19: a rapid prospective nationwide consensus study in Spain with 375 cases. *British Journal of Dermatology*.

[201] Fernández-Lázaro D., Garrosa M. (2021). Identification, mechanism, and treatment of skin lesions in COVID-19: a review. *Viruses*.

[202] Piccolo V., Neri I., Manunza F., Mazzatenta C., Bassi A. (2020). Chilblain-like lesions during the COVID-19 pandemic: should we really worry?. *International Journal of Dermatology*.

[203] Daneshgaran G., Dubin D. P., Gould D. J. (2020). Cutaneous manifestations of COVID-19: an evidence-based review. *American Journal of Clinical Dermatology*.

[204] Zhou M., Zhang X., Qu J. (2020). Coronavirus disease 2019 (COVID-19): a clinical update. *Frontiers of Medicine*.

[205] Zhang Y., Cao W., Xiao M. (2020). Clinical and coagulation characteristics in 7 patients with critical COVID-2019 pneumonia and acro-ischemia. *Zhonghua Xue Ye Xue Za Zhi*.

[206] Fernandez-Nieto D., Jimenez-Cauhe J., Suarez-Valle A. (2020). Comment on “Characterization of acute acro-ischemic lesions in non-hospitalized patients: a case series of 132 patients during the COVID-19 outbreak”. *Journal of the American Academy of Dermatology*.

[207] Marzano A. V., Genovese G., Fabbrocini G. (2020). Varicella-like exanthem as a specific COVID-19-associated skin manifestation: multicenter case series of 22 patients. *Journal of the American Academy of Dermatology*.

[208] Algaadi S. A. (2020). Urticaria and COVID-19: a review. *Dermatologic Therapy*.

[209] Jimenez-Cebrian A. M., Castro-Mendez A., García-Podadera B. (2021). Clinical manifestations of COVID-19 in the feet: a review of reviews. *Journal of Clinical Medicine*.

[210] Rusu L. C., Ardelean L. C., Tigmeanu C. V., Matichescu A., Sauciur I., Bratu E. A. (2021). COVID-19 and its repercussions on oral health: a review. *Medicina (Kaunas)*.

[211] Corchuelo J., Ulloa F. C. (2020). Oral manifestations in a patient with a history of asymptomatic COVID-19: case report. *International Journal of Infectious Diseases*.

[212] Sinjari B., Rexhepi I., Santilli M. (2020). The impact of COVID-19 related lockdown on dental practice in Central Italy-outcomes of A survey. *International Journal of Environmental Research and Public Health*.

[213] Mehic D., Adna M., Jusufovic R., Catic T. (2021). COVID-19 impact on access and performing dental care in Bosnia and Herzegovina. *Mater Sociomed*.

[214] Mac Giolla Phadraig C., van Harten M. T., Diniz-Freitas M. (2021). The impact of COVID-19 on access to dental care for people with disabilities: a global survey during the COVID-19 first wave lockdown. *Med Oral Patol Oral Cir Bucal*.

[215] Marouf N., Cai W., Said K. N. (2021). Association between periodontitis and severity of COVID-19 infection: a case-control study. *Journal of Clinical Periodontology*.

[216] Basso L., Chacun D., Sy K., Grosgogeat B., Gritsch K. (2021). Periodontal diseases and COVID-19: a scoping review. *European Journal of Dermatology*.

[217] Burton M. J., Clarkson J. E., Goulao B. (2020). Antimicrobial mouthwashes (gargling) and nasal sprays administered to patients with suspected or confirmed COVID-19 infection to improve patient outcomes and to protect healthcare workers treating them. *Cochrane Database of Systematic Reviews*.

[218] Suzuki J. B., Delisle A. L. (1984). Pulmonary actinomycosis of periodontal origin. *Journal of Periodontology*.

[219] Dziedzic A., Wojtyczka R. (2021). The impact of coronavirus infectious disease 19 (COVID‐19) on oral health. *Oral Diseases*.

[220] Wu C., Chen X., Cai Y. (2019). Risk factors associated with acute respiratory distress syndrome and death in patients with coronavirus disease 2019 pneumonia in wuhan, china.

